# SMAD4 variants and its genotype–phenotype correlations to juvenile polyposis syndrome

**DOI:** 10.1186/s13053-023-00267-z

**Published:** 2023-12-08

**Authors:** Kimberley Cao, John-Paul Plazzer, Finlay Macrae

**Affiliations:** 1 Department of Colorectal Medicine and Genetics, The Royal Melbourne Hospital, Parkville, VIC Australia; 2 Melbourne Medical School, University of Melbourne, Parkville, VIC Australia

**Keywords:** Juvenile polyposis syndrome, SMAD4 protein, Genotype–phenotype correlation, Gene analysis

## Abstract

**Background:**

Juvenile polyposis syndrome (JPS), a rare autosomal dominant syndrome, affects one per 100 000 births, increasing lifetime cancer risk by 9 – 50%. Around 40–60% of JPS cases are caused by disease-causing variants (DCV) in SMAD4 or BMPR1A genes, of which SMAD4 accounts for 20–30%.

**Objectives:**

To characterise genotype–phenotype correlations between sites and types of variants within SMAD4 to JPS phenotypes, to inform diagnosis, screening, and management of JPS.

**Search methods:**

Online search databases utilised included Ovid MEDLINE, Embase Classic + Embase and PubMed, using search terms classified by MeSH on Demand. Adjacency operators, word truncation and Boolean operators were employed. 110 articles were included in the review, collating 291 variants from the literature.

**Results:**

In SMAD4 + JPS patients, most variants are located around SMAD4’s MH2 domain (3’ end). Extracolonic involvement, massive gastric polyposis and a more aggressive phenotype have been associated with SMAD4 + JPS, predisposing to gastric cancer. This has contributed to an overall higher incidence of GI cancers compared to other genes causing JPS, with DCVs mostly all within the MH2 domain. Genetically related allelic disorders of SMAD4 also have variants in this region, including hereditary haemorrhagic telangiectasia (HHT) alongside SMAD4 + JPS, and Myhre syndrome, independent of JPS. Similarly, with DCVs in the MH2 domain, Ménétrier’s disease, hypertrophic osteoarthropathy and juvenile idiopathic arthritis have been seen in this population, whereas cardiac pathologies have occurred both alongside and independently of SMAD4 + JPS with DCVs in the MH1 domain.

**Conclusion:**

Truncating and missense variants around the MH2 region of SMAD4 are most prevalent and pathogenic, thus should undergo careful surveillance. Given association with extracolonic polyposis and higher GI cancer risk, endoscopic screening should occur more frequently and at an earlier age in SMAD4 + JPS patients than in patients with other causative genes, with consideration of Ménétrier’s disease on upper GI endoscopy. In addition, HHT should be evaluated within 6 months of diagnosis, alongside targeted clinical examination for extraintestinal manifestations associated with SMAD4 + JPS. This review may help modify clinical diagnosis and management of SMAD4 + JPS patients, and aid pathogenicity classification for SMAD4 DCVs through a better understanding of the phenotypes.

## Introduction

**Fig. 1:**
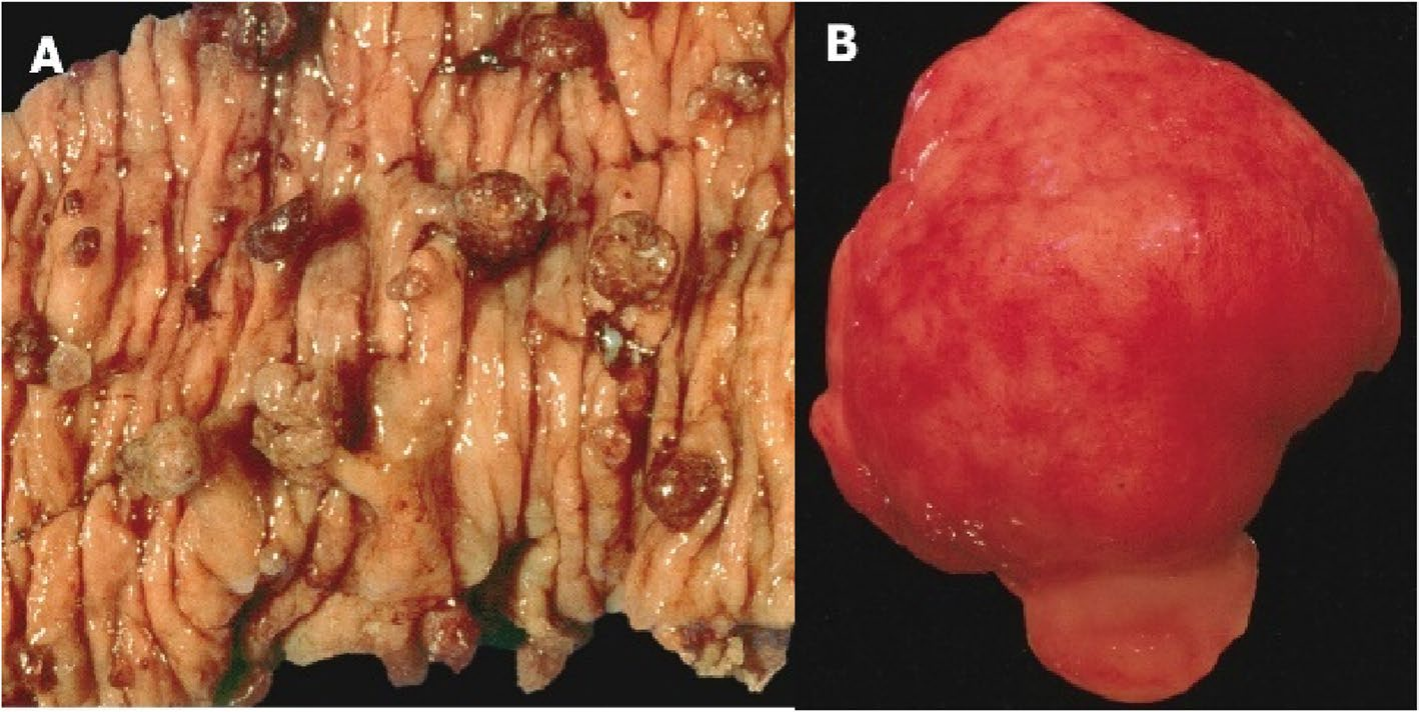
Macroscopic appearance of JPs. **A** Multiple pedunculated polyps with smooth surfaces post-bowel resection in a JPS patient. **B** JP from a patient with JPS, noted for its smooth surface [14]

**Fig. 2:**
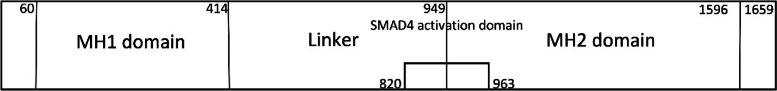
Structure of the SMAD4 gene, involving MH1 domain, linker domain and MH2 domain (self-made)

**Fig. 3:**
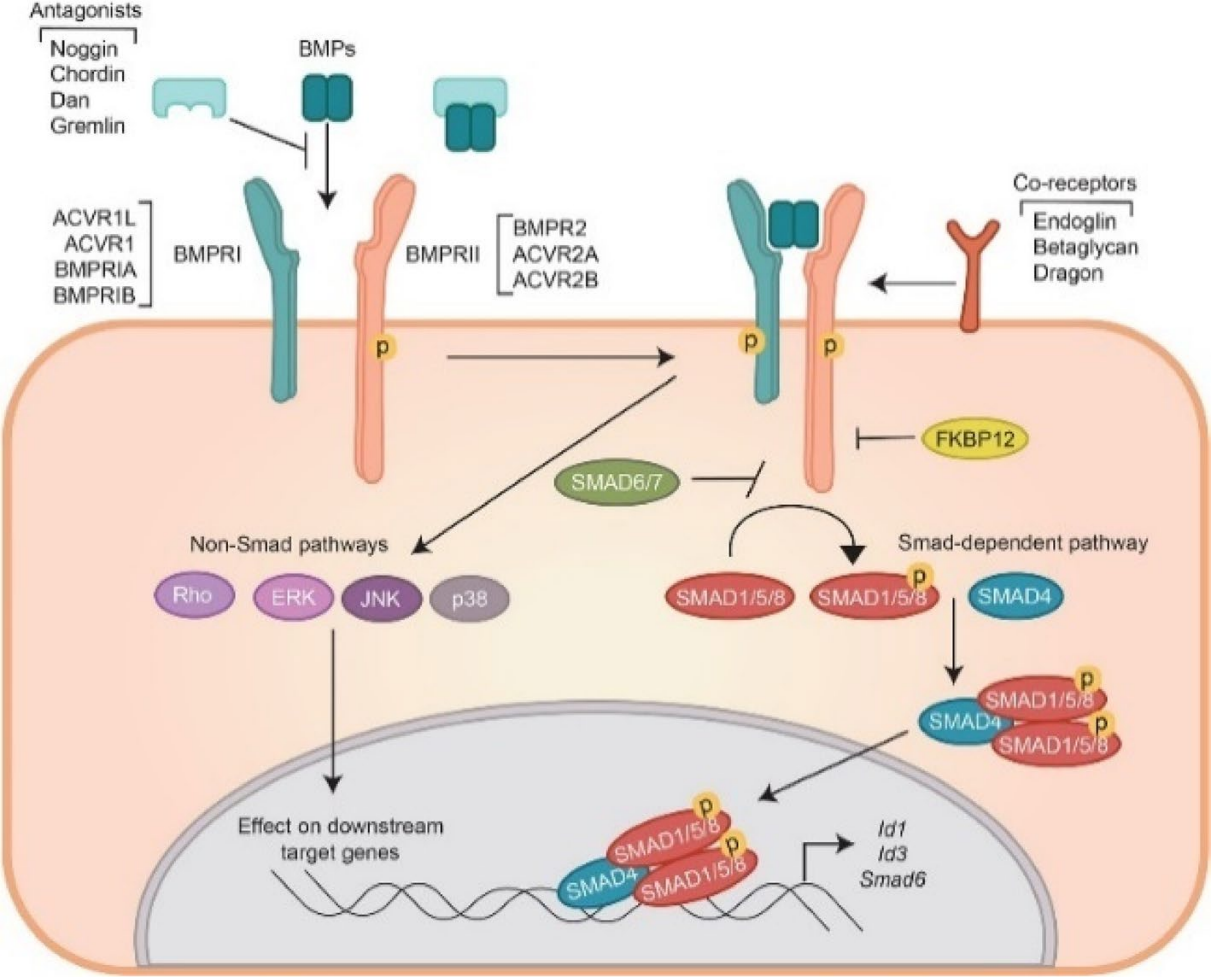
Illustration showing SMAD4’s (blue oval) involvement in the TGF-β and BMP pathways [39]

JPS is a rare autosomal dominant syndrome affecting one per 100 000 births, where 50 – 75% of affected patients have a positive family history [19, 26]. Hamartomatous polyps occur throughout the GIT, increasing cumulative lifetime risk of GI cancer by 9 – 50%, which is decreased through increased surveillance [65]. Such “juvenile” polyps (JP) are histologically described as having dense stroma with inflammatory infiltrate with mucus-filled cystic glands in the lamina propria (Figs. 1, 2, and 3).

Clinical diagnosis of JPS is confirmed if any one of the following 3 criteria are met, given there is absence of syndromic extra-intestinal features that define other hamartomatous polyposis syndromes:

1.  > 5 colonic hamartomatous polyps at one time or recurrent
2. Any number of hamartomatous polyps in a patient with family history of JPS
3. Extracolonic hamartomatous polyps (e.g., stomach, small bowel)

It is estimated that 40–60% of JPS cases are caused by DCVs in SMAD4 or BMPR1A genes, mostly consisting of missense, nonsense, deletions, and small insertions, together with large genomic deletions [4]. Germline SMAD4 DCVs have been observed in 20–30% JPS cases [3], similarly to BMPR1A, located at chromosome 10q23.2. PTEN DCVs have been sequenced in approximately 5% of JPS patients, near BMPR1A at chromosome 10q23.3, though confounds with Cowden syndrome. Finally, ENG DCVs have recently been associated with JPS, located at chromosome 9q34.1 with known associations to HHT [48, 77]. For example, Howe and colleagues [45] found ENG DCVs in 13/31 JPS patients without DCVs in SMAD4 or BMPR1A, whereas 2/11 JPS patients were ENG^+^ in Sweet and colleagues’ study [102]. Currently, > 50% of JPS cases have had causative genes described, all involved in the TGF-β signalling pathway, modulating colonic epithelial growth [38, 47].

The SMAD4 gene is located on chromosome 18q21.1 with 55 000 base pairs encompassing 11 exons, encoding a 551-amino acid protein. Functional domains include the MH1 domain, involved in DNA binding, a domain linking the two MH domains, and the MH2 domain which is involved in homodimerisation, heterodimerisation, and transcriptional activation and nuclear location of SMAD4 [48, 75].

Its protein product acts as an intracellular mediator to TGF-β and downstream bone morphogenetic protein (BMP) signalling, having a major role in colonic epithelial growth [21, 48]. Following activation of TGF-β, members of the SMAD family form a complex with SMAD4 via its COOH terminus. Mutations disrupting this complex formation result in loss of TGF-β signalling, thus partially explaining why most germline mutations of SMAD4 map to this domain [92]. Physiologically, these complexes are then transported to the nucleus, signalling growth inhibition. It is hypothesised that heterozygous loss-of-function (LOF) SMAD4 mutations may prevent formation of these complexes, thus promoting growth, resulting in cellular proliferation and subsequently neoplasia [39]. Potentially, DCVs in the linker region are less prominent, given this region is often deleted during alternative splicing.

Bosman’s hamartoma-adenoma-carcinoma sequence theory [13] postulates a “landscaper” defect, where SMAD4 mutations disrupt epithelial architecture, differentiation, and proliferation via altering the microenvironment. This pathway begins with JP formation, adenomatous change, dysplasia, then finally carcinoma [54, 55].

Current knowledge of genotype–phenotype correlations between SMAD4 variants and JPS is that individuals are more inclined to have upper gastrointestinal (UGI) polyposis and higher gastric cancer risk, as compared to BMPR1A [91]. HHT is exclusively seen within SMAD4 + JPS patients [8]. Thus, current treatment involves regular surveillance via routine endoscopy with polypectomy, together with monitoring and treatment of HHT manifestations [65].

Further detailed genotype–phenotype correlations between SMAD4 DCVs and JPS subtypes are yet to be fully described in this field of research. Better characterisation of these associations will help modify clinical diagnosis, screening, surveillance, and management of SMAD4 + JPS patients. In addition, this research will also aid pathogenicity classification for SMAD4 variants where phenotypic manifestations are incorporated into modified American College of Medical Genetics (ACMG) criteria, allowing, for example, segregation analyses to assist in classification.

This narrative review’s overarching research question is thus, in SMAD4 variant carriers, what are existing genotype–phenotype correlations relating to sites and types of variants within the gene, particularly focusing on phenotypes of JPS? Further from this, what implications will these correlations have on clinical management and for gene specific modifications to ACMG criteria?

## Methods

A literature search was performed on July 27, 2021, via three online search databases: Ovid MEDLINE, Embase Classic + Embase, and PubMed. Search strategy utilised key words surrounding the research question: SMAD4, JPS, and gene association studies, via MeSH on Demand.

Regarding JPS, key words included juvenile polyposis, intestinal polyposis, JPS and hamartomatous polyposis syndrome. Aliases of SMAD4 gene included SMAD family member 4, MADH4, DPC4, JIP, MYHRS, mothers against decapentaplegic homolog 4, deletion target in pancreatic carcinoma 4, MAD homolog 4, deleted in pancreatic carcinoma locus 4 and HSMAD4. To capture keywords related to genotype–phenotype association studies, words included genotype, gene, genome, phenotype, DNA, mutation, chromosome, variant, and variations of gene associated studies. Boolean operators including AND and OR were utilised, adjacency operators, as well as word truncation to enable different forms of words to be searched for simultaneously (Appendix A).

Specific inclusion criteria included English and human studies, retrospective and prospective gene studies, unique case reports and reviews which discuss JPS, SMAD4 DCVs and/or genotype–phenotype correlations. Exclusion criteria included any non-English and animal studies, non-significant case reports, and studies that did not mention juvenile polyposis syndrome nor SMAD4.

## Results

**Fig. 4:**
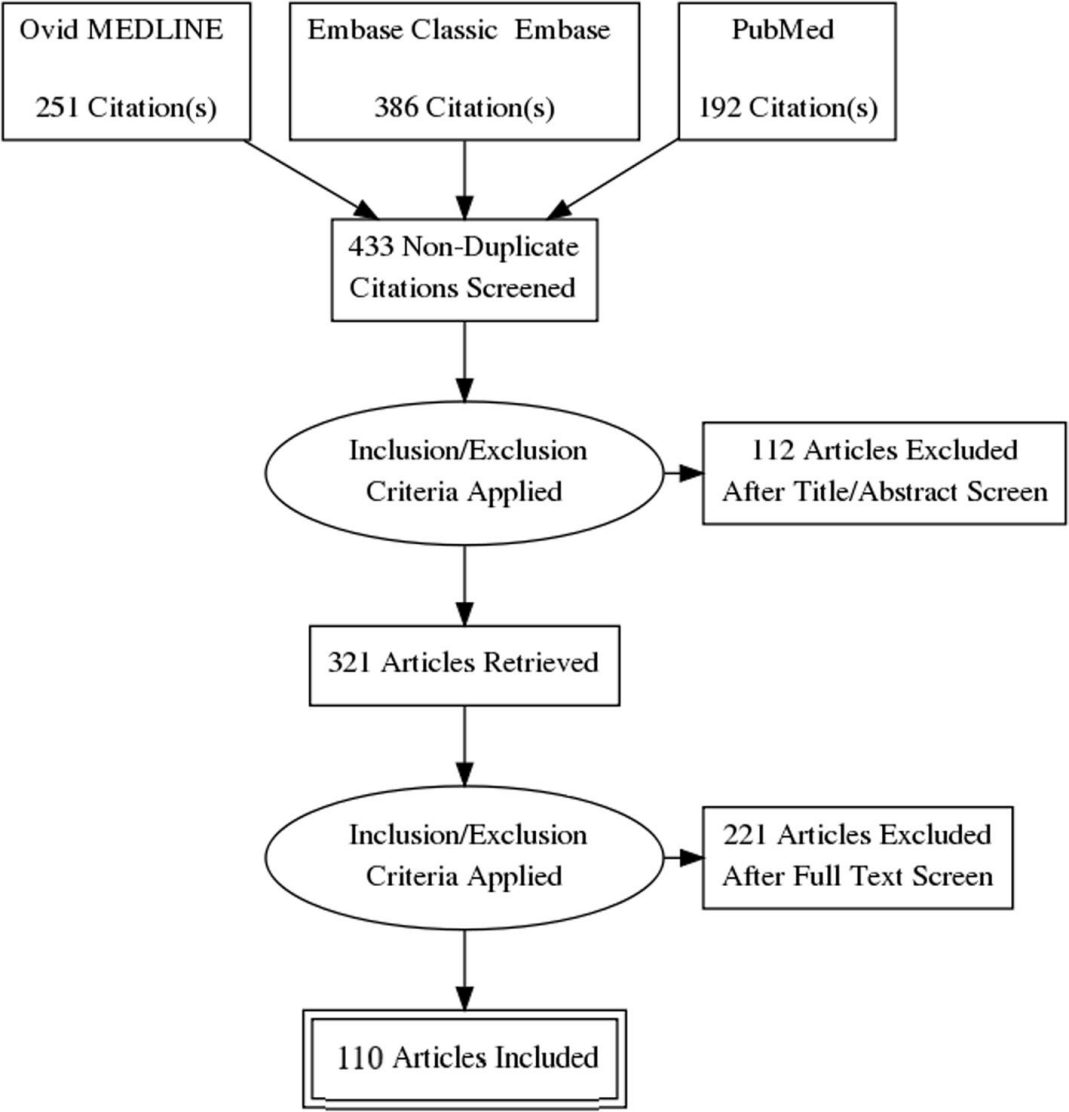
PRISMA Diagram presenting the review process for this narrative review (self-made)

**Fig. 5:**
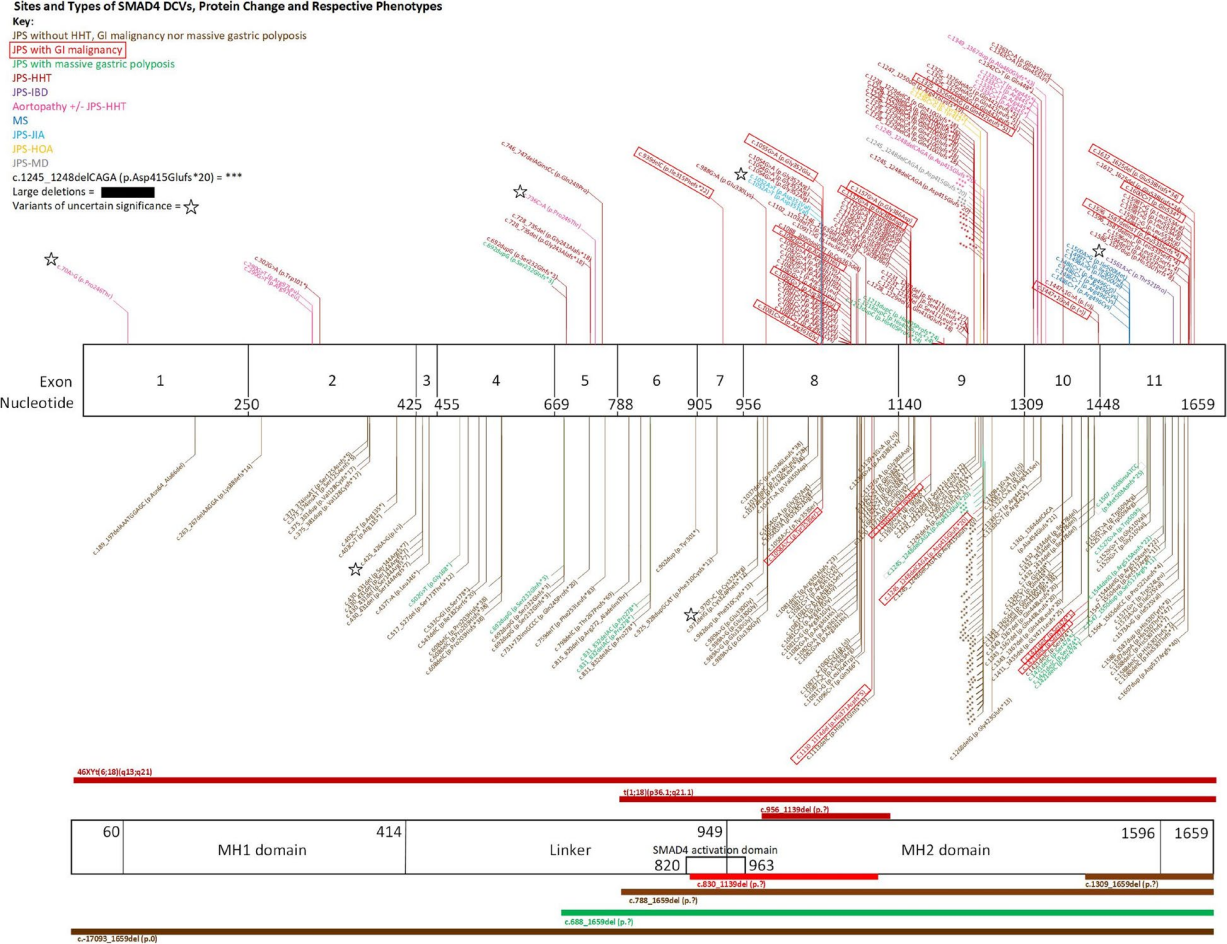
Sites and types of SMAD4 DCVs, protein change and phenotypes, self-made via Microsoft Visio. All 291 variants were collated from the literature. In the top section, SMAD4 gene structure is represented via its exons demarcated by nucleotide numbers. DCVs causing JPS phenotypes have been depicted with lines extending below the diagram in brown (JPS), green (massive gastric polyposis in JPS) and red (GI cancer in JPS). Above the illustrations represent extra-gastrointestinal phenotypes, including HHT (dark red), aortopathy (pink), IBD (purple), Myhre syndrome (dark blue), JIA (light blue), hypertrophic osteoarthropathy (yellow), Ménétrier’s disease (grey) and concurrent GI cancer boxed in bright red. In the bottom section, large deletions and chromosomal translocations are portrayed via solid lines, surrounding the SMAD4 gene, where JPS phenotypes are below, and extra-GI phenotypes are above. c.1245_1248delCAGA (p.Asp415Glufs*20), a highly prevalent DCV, is represented by asterisks (*). Variants of uncertain significance (VUS) are marked with adjacent stars, sourced from JPS registries and case reports, with pathogenicity reviewed by ClinVar

From this search, 829 studies were identified from three databases (Ovid MEDLINE = 251, Embase Classic + Embase = 386, PubMed = 192). 396 of these were duplicates, 30 were animal studies not excluded from search strategy, and 82 were considered irrelevant as they did not involve SMAD4, JPS or its causative genes, amounting to 321 studies. Full text screening thus isolated 110 papers, including narrative reviews, retrospective and prospective gene studies, together with pertinent case reports relevant to this review, pertaining to SMAD4 and its genotype–phenotype correlations to JPS and relevant conditions (Fig. 4). Results are tabulated in Fig. 5, Tables 1 and 2**,** and are further elucidated in the discussion.

**Table 1:** List of gene studies, including title, sample size, study design, results, and limitations

Author & year	Title	Sample size	Study design	Aims	Findings/results	Limitations
**Aretz et al. (2007) **[4]	High proportion of large genomic deletions and a genotype–phenotype update in 80 unrelated families with juvenile polyposis syndrome	80 patients, 65 of which were confirmed JPS, 15 suspected	Retrospective gene study	To characterise the frequency of large genomic deletions in SMAD4 and BMPR1A genes	Via gene sequencing, point mutations were identified in 30 patients (46%; SMAD4 = 17, BMPR1A = 13). Of SMAD4 point mutations, 11 led to truncated proteins (5 nonsense, 6 frameshift). 4 missense mutations were located in highly conserved AA positions (2 de novo, 1 mosaic). Large genomic deletions were found in 14% of all patients with typical JPS (SMAD4 = 6, BMPR1A = 3). From SMAD4, 4 had heterozygous deletion of all SMAD probes, the other 2 deleted coding exons from 6–11. SMAD4 mutation carriers had a significantly higher frequency of gastric polyposis (73%) than BMPR1A carriers (8%). All 7 cases of gastric cancer occurred in families with SMAD4 mutations. In 22% of the SMAD4 carriers, HHT was also diagnosed clinically (n = 5, 22%).	Small patient numbers, thus limited statistical analysis
**Aytac et al. (2014) **[5]	Oncologic outcomes and survival in juvenile polyposis syndrome with BMPR1A or SMAD4 mutation	35 JPS patients (SMAD4 n = 27, BMPR1A n = 8)	Prospective study, ~ 12 year follow-up	To evaluate oncologic outcomes in JPS patients with SMAD4 or BMPR1A gene mutations	Colonic phenotype (including rectal polyps) was similar between patients with SMAD4 and BMPR1A mutations, though SMAD4 mutations were associated with high gastric polyp numbers. Small bowel polyps diagnosed in 5 patients. No cancer in BMPR1A group, but 4 men with SMAD4 mutations developed cancer, wth a GI cancer risk of 11% (3/27). 2 patients with SMAD4 died during follow-up, all suggesting SMAD4 genotype is associated with more aggressive upper GI manifestations of JPS.	N/A
**Aytac et al. (2015) **[6]	Genotype-defined cancer risk in juvenile polyposis syndrome	35 JPS patients with mutations in either BMPR1A (8) or SMAD (27)	Prospective study	To investigate the impact of genotype on cancer risk and oncological phenotype in patients with JPS with a SMAD4 or BMPR1A gene mutation	Colonic phenotype was similar between patients with SMAD4 and BMPR1A mutations, where SMAD4 mutations were associated with larger gastric polyp numbers (14 with > 50 polyps). Small bowel juvenile polyps were diagnosed in 14 patients with SMAD4, and 2 with BMPR1A. No patient was diagnosed with cancer in the BMPR1A group, whereas four men with SMAD4 mutation developed GI or extra-intestinal cancer. The GI cancer risk in patients with JPS and SMAD4 mutation was 11%.	Retrospective nature of study and small patient numbers
**Barlas et al. (2012) **[8]	Follow-up of juvenile polyposis syndrome patients with BMPR1A or SMAD4 mutation	20 JPS patients (SMAD4 n = 15, BMPR1A n = 5)	Prospective study	To report clinical and natural history differences in JPS patients based on their underlying mutation	Of the 15 SMAD + ve JPS patients, 9 had a family history of JPS (while none of the BMPR1A + ve patients did). 10 of the 15 patients with SMAD4 mutations also had HHT. 7/15 had gastric polyps, where there were none in the BMPR1A group.	N/A
**Blatter et al. (2015) **[11]	Somatic alterations in juvenile polyps from BMPR1A and SMAD4 mutation carriers	25 polyps from 3 patients	Clinicopathological analysis	To evaluate the frequency and nature of the 2nd-hit mutations in juvenile polyposis in a series of 25 polyps from SMAD4 and BMPR1A mutation carriers, assessing their compartmental localisation	25 polyps from 3 patients were screened carrying either a pathogenic SMAD4 variant (c.1244-1246delACAG) or BMPR1A variant. No somatic alterations could be identified in the 14 SMAD4-related polyps. SMAD4 protein expression, however, was lost in 8 of 14 (57%) juvenile polyps. LOH was gene copy number neutral and had occurred in the epithelial compartment.	Nil mentioned
**Blatter et al. (2020) **[10]	Disease expression in juvenile polyposis syndrome: a retrospective survey on a cohort of 221 European patients and comparison with a literature-derived cohort of 473 SMAD4/BMPR1A pathogenic variant carriers	221 JPS patients from ten European centres from 126 kindreds 473 carriers of SMAD4/BMPR1A from the literature	Retrospective study Questionnaire-based data analysis	To gather detailed clinical and molecular genetic information on JPS disease expression to provide a basis for management guidelines and establish open access variant databases	Compared to BMPR1A carriers, SMAD4 carriers displayed anaemia twice as often (58% vs 26%), and exclusively showed overlap symptoms with HHT (32%) with an increased prevalence of gastric juvenile polyps (39% vs 13%). Cancer was reported in 15% of JPS patients, mainly in the colorectum (62%; SMAD4: 58%, BMPR1A: 88%) and the stomach (21%; SMAD4: 27%, BMPR1A: 0%). SMAD4 DCV carriers displayed a more severe gastric phenotype and HHT overlap phenotype. SMAD4 missense DCVs diplayed younger median age at diagnosis (10.5y) compared to frameshift (28.0y).	Possible ascertainment ± selection bias given retrospective study. Differences in patient data collection and medical record completeness may impact data quality and comparability. Potential underestimations of phenotypic features if not specifically searched for
**Bonjean et al. (2013)** [12]	Clinical expression of hereditary haemorrhagic telangiectasia and digestive lesion characteristics in patients with SMAD4 mutation	14 SMAD + ve HHT patients (out of 589 HHT patients)	Retrospective analysis	To define clinical expression of HHT and GI lesions in HHT patients with SMAD4 mutation	Of the 14 SMAD + ve HHT patients, symptoms included: epistaxis (13), telangiectasia (11), pulmonary venous malformations (9). More severe symptoms included diffuse/multiple PVMs causing hypoxemia (4) and severe hepatic AVM with high cardiac output (3/11). 11 had endoscopies, where 10 (91%) had digestive lesions, with 4 confirming a JP diagnosis—with 5 upper GI and 9 lower GI lesions.	Small cohort
**Calva-Cerqueira et al. (2009) **[20]	The rate of germline mutations and large deletions of SMAD4 and BMPR1A in juvenile polyposis	102 JPS patients	Retrospective gene study	To determine the overall prevalence of genetic changes of SMAD4 and BMPR1A by sequencing and by screening for larger deletions	Via gene sequencing, 20 JPS patients had point mutations of SMAD4, 22 of BMPR1A. By multiplex ligation-dependent sequencing, one proband had deletion of most of SMAD4, one of both BMPR1A & PTEN, one of the 5' end of BMPR1A & another at the 5' end of SMAD4. Overall prevalence of SMAD4/BMPR1A point mutations was 45%, with large deletions being less frequent, though can represent other heritable causes of JPS which should be screened for (e.g. promoter region, non-coding exons, introns, 3' untranslated region).	Nil mentioned
**Duan et al. (2019) **[29]	SMAD4 rare variants in individuals and families with thoracic aortic aneurysms and dissections	223 families with HTAD (heritable thoracic aortic disease)	Retrospective gene study	To characterise SMAD4 rare variants in patients with thoracic aortic aneurysm/dissection	A *SMAD4* heterozygous variant, c.290G > T, p.(Arg97Leu) was identified in a family with thoracic aortic disease and no evidence of HHT or JPS. In addition, two rare variants were identified in individuals with early age of onset of thoracic aortic dissection. These results suggest that SMAD4 rare missense variants can lead to thoracic aortic disease in individuals who do not have JPS or HHT. It is hypothesised that the missense variant in MH1 domain of SMAD4 leads to unstable protein and segregates with thoracic aortic disease in absence of JPS/HHT.	Additional studies needed to validate and determine the frequency of SMAD4 variants leading to thoracic aortic disease in absence of features of JPS/HHT
**Friedl et al. (1999) **[33]	Frequent 4-bp deletion in exon 9 of the SMAD4/MADH4 gene in familial juvenile polyposis patients	11 unrelated patients with familial JP	Retrospective gene study	To evaluate the proportion of SMAD4 mutations in familial juvenile polyposis (FJP)	Through gene sequencing, 3/11 patients had SMAD 4 mutations. A 4-bp deletion (1372-1375delACAG) in exon 9 was observed in two unrelated patients. A novel mutation was found also, 959-960delAC at codon 277, exon 6.	Nil mentioned
**Friedl et al. (2002) **[34]	Juvenile polyposis: massive gastric polyposis is more common in MADH4 mutation carriers than in BMPR1A mutation carriers	29 JPS patients	Retrospective gene study	To assess possible genotype–phenotype correlations in JPS	Germline MADH4 mutations were found in 24% cases (7) and BMPR1A in 17% (5). Prevalence of massive gastric polyposis was observed in patients with MADH4 mutations (4/7), when compared to BMPR1A. Of the SMAD4 mutations, all but one mutation resulted in a truncated protein, where 6/7 were located within the highly conserved MH2-region.	Not mentioned
**Gallione et al. (2004) **[36]	A combined syndrome of juvenile polyposis and hereditary haemorrhagic telangiectasia associated with mutations in *MADH4* (*SMAD4)*	7 unrelated families with JP-HHT (13 total patients)	Retrospective gene study	To investigate the underlying aetiology of JP-HHT syndrome, via characterising individuals with this phenotype clinically and molecularly	Mutations in MADH4 were identified in all affected individuals with the combined phenotype. Mutations included 4 missense, 1 nonsense, and 2 frameshift mutations in exons 8, 9, 11 of MADH4 (i.e. COOH terminus). 3 had de novo mutations in MADH4, also exhibiting the combined syndrome. Individuals with MADH4 mutation showed phenotypes of both disorders, phenotypes including: multiple juvenile polyps, plus telangiectases (9), epistaxis (9), pulmonary AVM (7), clubbing/osteoarthropathy (7)	Nil mentioned
**Gallione et al. (2006) **[37]	SMAD4 mutations found in unselected HHT patients	30 unrelated HHT patients without apparent history of JP	Retrospective gene study	To characterise the frequency of SMAD4 mutations in HHT patients without a history of JP	10% (n = 3) harboured mutations in SMAD4, similar to those found in other patients with JP-HHT syndrome—found in the COOH terminus of SMAD4, where all mutations in previously reported JP-HHT cases have been identified.	Nil mentioned
**Gallione et al. (2010) **[35]	Overlapping spectra of SMAD4 mutations in juvenile polyposis and JP-HHT syndrome	19 JP-HHT patients, 15 SMAD4 mutations	Retrospective gene study	To evaluate whether the originally observed genotype:phenotype correlation is valid (i.e. mutations clustered within MH2 domain)	Although SMAD4 mutations in JP-HHT have a tendency to cluster in the MH2 domain (13/15), mutations in other parts of the gene also cause the combined syndrome (2/15) i.e. any mutation in SMAD4 can cause JP-HHT. Thus, any patient who tests positive for any SMAD4 mutation should be considered at risk for the combined JP-HHT syndrome & be monitored accordingly.	Nil mentioned
**Gonzalez et al. (2017) **[40]	Massive gastric juvenile-type polyposis: a clinicopathological analysis of 22 cases	22 patients with abundant juvenile-type or hyperplastic-like polyps, 14 diagnosed with JPS	Clinicopathological analysis	To evaluate the clinicopathological features of 22 patients with gastric juvenile-type or hyperplastic-like polyps	SMAD4 immunohistochemical staining showed patchy loss in polyps from 19 of 20 cases tested. 5 of 6 patients tested had a germline SMAD4 mutation. Massive gastric juvenile-type polyposis can occur in patients with and without known JPS, where SMAD4 mutation appears to be the greatest risk factor for upper tract involvement.	Not mentioned
**Handra-Luca et al. (2005) **[41]	Vessels' morphology in SMAD4 and BMPR1A-related juvenile polyposis	42 JPS patients	Retrospective gene study	To identify specific gene defects in those with JPS, which may be useful in predictive genetic testing and subsequent preventive screening & treatment	Mutations found in 14 patients, 9 in SMAD4 (21.5%), 5 in BMPR1A (11.9%). All but one were truncating mutations, and the remaining were point mutations. Only patients with SMAD4 mutations harboured carcinoma lesions (5/9). Malformative vessels were present in all SMAD4-related polyps when the mutation involved codons prior to position 423. Lack of SMAD protein was observed in 13 cases of polyps, 6 of which harboured germline SMAD4 mutation. SMAD4 germline mutations are responsible for a more aggressive digestive phenotype in JPS patients—associated with low-grade adenoma, high-grade adenoma/adenocarcinoma, upper GI location, and presence of malformative vessels within the polyp stroma (in absence of obvious HHT).	Nil mentioned
**Hattem et al. (2008) **[104]	Large genomic deletions of SMAD4, BMPR1A and PTEN in juvenile polyposis	29 JPS patients	Retrospective gene study	To perform a comprehensive genetic analysis of SMAD4, BMPR1A, PTEN and ENG, to address whether large genomic deletions of any known JPS genes may cause JPS	Of the 27 patients, 6 had SMAD4 mutations (22%), 3 BMPR1A (11%). Of the SMAD4 mutations, there were 2 missense mutations (exon 8), one nonsense (exon 9), 1 bp deletion (exon 8), 25 bp deletion (exon 10), and a single base pair duplication (exon 11).	Not mentioned
**Heald et al. (2015) **[43]	Prevalence of thoracic aortopathy in patients with Juvenile Polyposis Syndrome-Hereditary Hemorrhagic Telangiectasia due to *SMAD4*	26 HHT patients / carriers of pathogenic mutations including SMAD4 (n = 16)	Retrospective chart review	To determine the prevalence of thoracic aortopathy in a JPS-HHT cohort	All six patients had SMAD4 mutations and JPS, who had aortopathy ranging from mild dilatation to aortic dissection (6/16 with SMAD4 mutation, 38%). This suggests aortopathy could be part of the spectrum of SMAD4-induced HHT manifestations.	Small numbers in cohort. Overrepresentation of patients with SMAD4 mutations given recruitment method
**Howe et al. (1998) **[47]	Mutations in the SMAD4/DPC4 Gene in Juvenile Polyposis	9 unrelated JP patients	Retrospective gene study	To evaluate the proportion and nature of SMAD4 mutations in patients with JPS	Of the 9 patients, 3 had a similar 4-bp deletion in exon 9 (codon 414–416, frameshift resulting in premature stop codon), one with 2-bp deletion in exon 8 (exon 348, premature stop codon), and one with 1-bp insertion resulting in frameshift and premature stop codon.	
**Howe et al. (1999)** [46]	Direct genetic testing for SMAD4 mutations in patients at risk for juvenile polyposis	Two large JP families 55 members, 18 with JP	Retrospective gene study	To define the role of genetic testing in the clinical management of patients with juvenile polyposis	All 18 affected family members (18/55), had a 4-bp deletion in exon 9 of the SMAD4 gene, leading to frameshift and premature stop codon at codon 434. In 30 patients at risk for JP, 17 previously had -ve endoscopic screening, while 13 had never been screened. 5 had inherited germline SMAD4 mutations, 2 with hematochezia without having been screened, and the other 3 were asymptomatic. This illustrates poor compliance with screening measures.	Nil mentioned
**Howe et al. (2004) **[48]	The prevalence of MADH4 and BMPR1A mutations in juvenile polyposis and absence of BMPR2, BMPR1B and ACVR1 mutations	77 JPS patients	Retrospective gene study	To determine the prevalence of mutations in MADH4 and BMPR1A in a large number of JP patients	Germline MADH4 mutations were found in 18.2% cases, 20.8% for BMPR1A (n = 77). No mutations found in BMPR1B, BMPR2 or ACVR1. Of the SMAD4 mutations, 8 were deletions (resulting in premature stop codons) and 6 were substitutions (nonsense, missense), all distributed across six of the 11 exons.	Not mentioned
**Howe et al. (2007)** [45]	ENG mutations in MADH4/BMPR1A mutation negative patients with juvenile polyposis	31 patients with JPS without mutations in SMAD4 or BMPR1A	Retrospective gene study	To examine the prevalence of ENG mutations in patients meeting diagostic criteria of JP who did not have germline mutations of SMAD4 or BMPR1A	Via gene sequencing, 13/31 of the patients had mutations in ENG. They had no clinical manifestations, family history of HHT, upper GI polyps. Mean age of JP diagnosis was 7.4y (compared to 14.4y for those without ENG mutations).	Limited sample size
**Jelsig et al. (2016)** [55]	JP-HHT phenotype in Danish patients with SMAD4 mutations	14 patients with SMAD4 mutation	Retrospective study	To describe the clinical characteristics of 14 patients with SMAD4 mutations	11 of 14 patients fulfilled criteria for JPS. 8 patients were screened for HHT symptoms, and 7 fulfilled the criteria for diagnosis. Thus, SMAD4 mutation carriers have symptoms of both HHT and JPS, with the frequency of PAVM and gastric involvement with polyps being higher than in patients with HHT/JPS not caused by the mutation.	Retrospective study, small patient numbers
**Jones et al. (2017) **[56]	SMAD4 mutation hotspot analysis and concomitant key cancer-related gene mutation profile in a large cohort of colorectal adenocarcinoma using next generation sequencing approach	242 CRC patients	Retrospective gene study	To evaluate the role of SMAD4 in a cohort of patients with CRC, utilising next-generation sequencing and immunohistochemistry	Frequency of SMAD4 mutations was 10.3% (25/242). Hotspot analysis for SMAD4 shows 40% (10/25) harbor a genetic alteration at the common codon 361, 88% (22/25) have missense mutations, 8% (2/25) have frameshift mutations, and one has a nonsense mutation. One case shows two point mutations at codons 352 and 523. Among the SMAD4-mutant CRC concomitant mutations include KRAS 52% (13/25), BRAF 16% (4/25), NRAS 4% (1/25), p53 44% (11/25), and PIK3CA 12% (3/25). SMAD4-mutant CRC lost expression of one or more DNA mismatch repair proteins in 16% (4/25). These tumors localized with 36% (9/25) in the right colon, 60% (15/25) in the left colon. Morphologic analysis demonstrates 48% (12/25) were moderately to poorly differentiated (high-grade) with 68% (17/25) either stage III or IV at the time of diagnosis, and 36% (9/25) demonstrate mucinous features.	Nil mentioned
**Kamil et al. (2012) **[58]	Colonic dysplasia and malignancy in patients with SMAD4 mutation-associated juvenile polyposis-hereditary hemorrhagic telangiectasia	15 JP-HHT patients	Retrospective review	To review the rate and types of dysplastic and malignant lesions in the JP-HHT phenotype	Of the 15 JP-HHT patients, the majority of the patients had only few juvenile polyps detected (3–5 polyps), but one patient had multiple (> 100 polyps). 6/15 patients developed dysplastic and malignant colonic lesions; 26 dysplastic lesions and 2 adenocarcinomas were identified at a mean patient age of 27.5 years. Four patients had lesions developing within juvenile polyps, including low grade and high grade dysplasia and signet ring carcinoma, the remainder of the dysplastic lesions were tubular or villous adenomas. Two patients developed invasive adenocarcinoma one year after dysplastic lesions were identified on colonoscopy.	Small patient numbers, thus limited statistical analysis
**Karlsson & Cherif (2018) **[60]	Mutations in the ENG, ACVRL1, and SMAD4 genes and clinical manifestations of hereditary haemorrhagic telangiectasia	21 patients with HHT	Retrospective. Single-centre study	To evaluate whether ENG, AVRL1, and SMAD4 genes were associated with different phenotypes in HHT	Of the 21 patients, 2 had mutations in SMAD4 and had the overlapping JPS-HHT syndrome. Both displayed a full range of HHT clinical features, as well as GI polyps, with one having colorectal cancer and the other having thyroid cancer.	Nil mentioned
**Latchford et al. (2012) **[65]	Juvenile polyposis syndrome: A study of genotype, phenotype, and long-term outcome	44 JPS patients	Retrospective review	To review clinical features, genetic mutations, and long-term outcome data in patients with juvenile polyposis syndrome	Out of the 31 JPS patients who underwent genetic testing, 19 had SMAD4 mutation (43.1%) and 9 had BMPR1A (20.5%). All patients with upper GI disease had SMAD4 mutations. Where germline mutation was known, all patients with telangiectasia had SMAD4 mutation, supporting JPS-HHT overlap, with a prevalence of 21% within the study cohort.	Retrospective review, cohort size (though good for a rare condition)
**Lawless et al. (2017) **[66]	Massive gastric juvenile polyposis: a clinical pathologic study using SMAD4 immunohistochemistry	9 cases of massive gastric juvenile polyposis	Clinicopathological analysis	To evaluate the clinicopathological features of 9 patients with gastric juvenile polyposis	6 out of 9 patients had loss of SMAD4 immunoreactivity, thus subject to severe bleeding and hypoproteinemia, as well as developing severe dysplasia or adenocarcinoma. Thus, SMAD4 immunohistochemistry is a helpful diagnostic test in suspected JPS involving the stomach.	No details of mutational testing available for comparison
**MacFarland et al. (2019) **[71]	Clinical presentation and disease progression in juvenile polyposis syndrome patients with and without a mutation in SMAD4 or BMPR1A	60 JPS patients	Cross-sectional analysis	To understand the potential differences in the clinical presentation and outcomes of patients with or without a known causative gene mutation	Of the 60 patients, in the pediatric cohort, 7 patients (17%) had a mutation in BMPR1A (n = 6) or SMAD4 (n = 1); in the adult cohort, 15 patients (79%) had a mutation in BMPR1A (n = 3) or SMAD4 (n = 12). Rate of SMAD4/BMPR1A mutation is lower in a paediatric cohort than adult. Presence of a mutation in SMAD4/BMPR1A is associated with a more severe course of disease, given all those requiring gastrectomy ± colectomy (n = 8), and all those who developed GI cancer (n = 3) had either mutation.	Small cohort
**MacFarland et al. (2021) **[72]	Phenotypic differences in Juvenile Polyposis Syndrome with or without a disease-causing SMAD4/BMPR1A variant	118 JPS patients	Retrospective study	To characterise the phenotype of DCV-negative JPS and compare it with DCV-positive JPS	Of the included JPS patients, 54 (46%) had mutations in SMAD4 (27) and BMPR1A (27). SMAD4 carriers were more likely to have a family history of JPS and required gastrectomy.	Data was collected from multiple different centres with differing levels of data granularity, limiting analysis of certain potential endpoints
**McDonald et al. (2020) **[75]	SMAD4 mutation and the combined juvenile polyposis and HHT syndrome: a single centre experience	22 patients with combined JP-HHT syndrome with SMAD4 mutation	Retrospective study, case series	To describe the phenotype and clinical outcomes of patients with genetically confirmed JP-HHT combined syndrome	All 22 patients had JPS-HHT combined phenotype with SMAD4 mutation. 77% had prior episode of epistaxis, 55% skin telangiectasia, 60% with visceral AV malformations. 82% had a family history (FHx) of HHT. Lower GI polyps found in 85% patients, upper GI in 68%, mainly in the stomach (10/15) and duodenum (5/15). FHx of polyps and CRC in 91% and 54% respectively.	Retrospective analysis, single centre experience
**Ngeow et al. (2013) **[77]	Prevalence of Germline *PTEN, BMPR1A, SMAD4, STK11,* and *ENG* Mutations in Patients With Moderate-Load Colorectal Polyps	603 patients with > 5 GI polyps with > 1 hamartomatous or hyperplastic polyp	Prospective, referral-based study	To determine prevalnce of hamartomatous polyposis-associated mutations in the susceptibility genes PTEN, BMPR1A, SMAD4, ENG and STK11	Of 603 patients, 21 had mutations in SMAD4 (3.5%) out of 77 who were mutation-positive. SMAD4 mutations were more commonly seen in patients with unexplained polyps if <40y and no FHx of CRC, and in patients with a positive FHx of GI polyps. Of 69 who met clinical criteria for JPS, 13 had germline SMAD4 mutations (18.8%).	Nil mentioned
**O'Malley et al. (2011)** [80]	The prevalence of hereditary hemorrhagic telangiectasia in juvenile polyposis syndrome	46 patients with JP	Retrospective cohort study	To determine the prevalence and clinical manifestations of hereditary hemorrhagic telangiectasia in JP SMAD4 + ve patients	SMAD4 mutations found in 21 patients—77% of mutations were in the MH2 domain of the gene between exons 8 and 11 (3'-located). 81% of SMAD + ve patients had HHT (17/21), with 14% suspected to have it (3/21). Epistaxis and asthma were the most common symptoms. 17 of the patients underwent HHT screening, with 16/17 meeting criteria for HHT diagnosis, and one suspected with 2 manifestations. 71% epistaxis, 57% telangiectasia, 86% visceral AVM, 81% pulmonary AVM (13/16).	Single, tertiary referral centre
**Pyatt et al. (2006) **[86]	Mutation screening in JPS	70 patients referred for JPS gene testing given family and medical history	Retrospective gene study	To describe experiences in laboratory after 3 years of molecular diagnostic screening for JPS	18.6% had mutations in MADH4, 11.5% in BMPR1A. Most MADH4 mutations were clustered towards 3' portion of the gene with 9 of 13 located in the MH2 domain of the protein. Small deletions were the most common type (> 50%).	Nil mentioned
**Sayed et al. (2002) **[91]	Germline SMAD4 or BMPR1A mutations and phenotypes of juvenile polyposis	54 patients with JP	Retrospective gene study	To determine the differences in phenotype of patients with SMAD4 or BMPR1A compared to those without these mutations	Of the 54 patients, 9 had germline SMAD4 mutations, 13 had BMPR1A mutations, and 32 had neither (59%). No significant differences were observed between SMAD4 & BMPR1A, apart from FHx of upper GI involvement. There was a higher prevalence of familial cases, > 10 lower GI polyps and frequency of GI cancer amongst mutation + ve patients compared with mutation -ve patients. Age of LGI polyposis diagnosis, FHx of upper GI polyps and FHx of cancer were significantly different between SMAD + ve and mutation -ve patients.	Nil mentioned
**Schwenter et al. (2012)** [94]	Juvenile polyposis, hereditary hemorrhagic telangiectasia, and early onset colorectal cancer in patients with *SMAD4* mutation	358 patients (HHT n = 332, JP n = 26)	Prospective study	To describe the phenotype of patients with JP-HHT and SMAD4 mutations, and to compare this phenotype with HHT or JP with mutations other than SMAD4	14 patients were identified with SMAD4 mutation, 10 met the criteria for both JP and HHT (71%). 57% presented with a haemorrhagic episode, 57% had abnormal echocardiography. Patients with SMAD4 mutations had 100% penetrance of the polyposis phenotype. All patients with JP and SMAD4 mutation had features of HHT. Three JP-HHT patients developed early onset CRC. JP-HHT patients with SMAD4 mutation had a significantly higher rate of anaemia than HHT patients with mutations other than SMAD4.	Not mentioned
**Suppressa et al. (2018) **[101]	Severe pulmonary involvement of SMAD4-mutated patients with juvenile polyposis/hereditary hemorrhagic telangiectasia combined syndrome	5 SMAD4 + ve patients	Cross-sectional prospective survey	To describe clinical pulmonary features of patients affected by JP/HHT and confirmed mutations in SMAD4, and compare lung AVM features with HHT1 + 2 patients	All 5 patients had pulmonary AVM and GI polyps. Silent hepatic involvement in 4/5. Clinically overt manifestations secondary to PAVMs was reported by 4/5 patients including hypoxaemia, digital clubbing, brain abscess/stroke. JP-HHT patients had significantly higher prevalence of complex PAVMs (compared to HHT1/2 patients).	N/A
**Sweet et al. (2005) **[102]	Molecular classification of patients with unexplained hamartomatous and hyperplastic polyposis	49 unrelated patients with multiple hamartomatous or hyperplastic polyps	Prospective gene study	To classify patients with unexplained hamartomatous or hyperplastic/mixed polyps by extensive molecular analysis in context of histopathology results	Of the 49 patients, 11 (22%) had germline mutations. 14 of these patients had juvenile polyposis, 2 of which had mutations in ENG (associated with HHT) with early-onset disease. 1 had an SMAD4 mutation, and 1 had a hemizygous deletion involving PTEN and BMPR1A. Thus, more extensive analysis of the known susceptibility genes is indicated.	Limited sample size
**van Hattem et al. (2011) **[105]	Histologic variations in juvenile polyp phenotype correlate with genetic defect underlying juvenile polyposis	39 JPS patients (90 polyps): 8 patients (21 polyps) with SMAD4 DCVs, 6 patients (44 polyps) with BMPR1A DCVs	Clinicopathological analysis	To compare the histologic phenotype of juvenile polyps with a SMAD4 or BMPR1A germline mutation and sporadic juvenile polyps	Juvenile polyps with a SMAD4 germline mutation were predominantly type B (crypt-stroma ratio > = 1.00; epithelial), whereas type A (crypt-stroma ratio < 1.00; classic, stromal juvenile polyp) was more common among juvenile polyps with a BMPR1A germline mutation. Dysplasia was equally common in JPS polyps with either a SMAD4 or BMPR1A germline mutation, where the adenoma-carcinoma sequence does not seem to play a distinct role.	Limited polyp numbers
**Wain et al. (2014) **[106]	Appreciating the broad clinical features of SMAD4 mutation carriers: a multicenter chart review	34 JPS patients of 20 families	Retrospective gene study	To understand the spectrum and extent of clinical findings in SMAD4 carriers	Of the 34 patients with SMAD4 mutations, 21% had features of a connective tissue defect, including enlarged aortic root (n = 3), aortic and mitral valve insufficiency (n = 2), aortic diseection (n = 1), retinal detachment (n = 1), brain aneurysms (n = 1), and lax skin and joints (n = 1). Juvenile polyposis specific findings were mostly uniformly present, where 30/31 (97%) patients had colonic polyps (pan-colonic) of variable histology and number. 11/28 had small bowel polyps (39%). 21/31 (68%) had gastric polyps, where 15/31 (48% patients) had extensive gastric polyposis. 9/34 had neoplasms, where 3/34 were CRC, 1/34 pancreatic cancer. HHT features were also prominent among the group, with 19/31 (61%) having epistaxis, 15/31 (48%) with telangiectases, 6/16 with liver AVMs, 1/26 with brain AVM, 9/17 with pulmonary AVM, and intrapulmonary shunting (14/23). SMAD4 carriers should be managed for JP & HHT, where connective tissue abnormalities are an emerging component.	Small sample size, young ages of some individuals, incomplete screening for all findings of interest in some individuals
**Woodford-Richens et al. (2000) **[108]	Analysis of genetic and phenotypic heterogeneity in juvenile polyposis	56 JPS patients where 47 were found from 15 families, and 9 were sporadic	Retrospective gene study	To describe the clinical features of JPS patients. To determine contribution of DPC4 mutations to JPS. Assess existence of any associations between germline mutations and clinical features. Determine proportion of JPS cases caused by as yet unidentified genes	5 germline DPC4 mutations were identified (n = 24). i.e. around 21%. Three of these were deletions ranging in size from two to 11 base pairs in exons 1, 4, and 11. One of the mutations was a single base substitution creating a stop codon in exon 10. The fifth mutation was a missense mutation in exon 8	Not mentioned

**Table 2:** Tabulated form of SMAD4 variants and associated phenotypes, where families are bolded

**Author & year**	Location	Type of mutation	SMAD4 nucleotide mutation	SMAD4 predicted protein change	ClinVar Class	Dx Age	Phenotype	Extra-gastrointestinal phenotypes
**Schwenter et al. (2012)** [94]	Exon 9; MH2 domain	Substitution, missense	c.1146C > A	p.(His382Gln)	Unclassified	1	JPS with multiple colorectal hamartomatous polyps and duodenal adenomatous polyps	HHT: Epistaxis, telangiectases, lung AVMs
	Intron 12; MH2 domain	Intronic	c.1447 + 1G > A	p.( =)	Pathogenic	28	JPS with FHx, multiple rectal hamartomatous polyps and gastric hyperplastic polyps; diagnosis of CRC	HHT: Epistaxis, telangiectases
	Exon 8; MH2 domain	Substitution, missense	c.1082G > T	p.(Arg361Leu)	Pathogenic	57	JPS with multiple colorectal hyperplastic polyps and few gastric hamartomatous polyps	HHT: Epistaxis, telangiectases, lung AVMs, cyanosis, digital clubbing, stroke
	Exon 8; MH2 domain	Substitution, missense	c.1082G > A	p.(Arg361His)	Pathogenic	34	JPS with FHx and multiple colorectal hamartomatous polyps	HHT: Epistaxis, telangiectases, lung AVMs
	Exon 11; MH2 domain	Deletion-insertion, frameshift	c.1596_1597delinsT	p.(Leu533Serfs*4)	Pathogenic	20	JPS with 10 colorectal polyps and 4 gastric polyps; diagnosis of CRC	HHT: Epistaxis, telangiectases, lung/liver AVMs
	Exon 5; Linker domain	Deletion, frameshift	c.728_735del	p.(Gly243Alafs*18)	Pathogenic	17	JPS with FHx and 20 colorectal hamartomatous polyps	HHT: Telangiectases, lung AVMs, cyanosis
	Exon 9; MH2 domain	Deletion, frameshift	c.1231_1232del	p.(Ser411Leufs*17)	Pathogenic	22	JPS with 28 colorectal polyps and multiple gastric hyperplastic polyps	HHT: Epistaxis, telangiectases, cyanosis
	Exon 8; MH2 domain	Substitution, miisense	c.1082G > A	p.(Arg361His)	Pathogenic	27	JPS with multiple colonic hamartomatous polyps and multiple duodenal adenomatous polyps	HHT: Epistaxis, telangiectases, lung AVMs, stroke, cyanosis
	Exon 9; MH2 domain	Deletion, frameshift	c.1231_1232del	p.(Ser411Leufs*17)	Pathogenic	31	JPS with FHx	HHT: telangiectases
	Exon 5; Linker domain	Substitution, missense	c.746_747delAGinsCC	p.(Gln249Pro)	Unclassified	10	JPS with FHx	HHT: Epistaxis, telangiectases
	Exon 8; MH2 domain	Substitution, missense	c.1082G > T	p.(Arg361Leu)	Pathogenic	12	JPS with FHx and 6 colorectal polyps	HHT: Epistaxis, lung AVMs
	Exon 5; Linker domain	Deletion, frameshift	c.728_735del	p.(Gly243Alafs*18)	Pathogenic	N/A	JPS with FHx, multiple colorectal hyperplastic polyps	HHT: Epistaxis
	Intron 12; MH2 domain	Intronic	c.1447 + 1G > A	p.( =)	Pathogenic	10	JPS with FHx and 9 colorectal juvenile polyps	HHT: Epistaxis, lung AVMs
**Ngeow et al. (2013) **[77]	Exon 6; SMAD4 activation domain	Deletion	c.(787 + 1_830)_(1139 + 1_1140-1)del	p.?	Unclassified	59	Juvenile; adenomatous polyps; diagnosis of CRC	
	Exon 10; MH2 domain	Deletion	c.1309-?_1659 + ?del	p.?	Unclassified	17	Juvenile polyps	
	Exon 3; Linker domain	Deletion, frameshift	c.430_431del	p.(Ser144Argfs*7)	Pathogenic	27	Juvenile polyps	
	Exon 3; Linker domain	Deletion, frameshift	c.430_431del	p.(Ser144Argfs*7)	Pathogenic	44	Adenomatous; hamartomatous polyps	
	Exon 6; Linker domain	Deletion, frameshift	c.798del	p.(Thr267Profs*69)	Unclassified	43	Juvenile; adenomatous polyps	
	Exon 6; SMAD4 activation domain	Insertion	c.902dup	p.(Tyr301*)	Unclassified	47	Juvenile; hyperplastic polyps	
	Exon 8; MH2 domain	Substitution, missense	c.1049 T > A	p.(Val350Asp)	Unclassified	9	Juvenile polyps	
	Exon 8; MH2 domain	Substitution, missense	c.1087 T > C	p.(Cys363Arg)	Pathogenic	2	Juvenile polyps	
	Exon 9; MH2 domain	Substitution, nonsense	c.1193G > A	p.(Trp398*)	Pathogenic	37	Juvenile; adenomatous polyps; diagnosis of gastric cancer	
	Exon 9; MH2 domain	Deletion, frameshift	c.1231_1232del	p.(Ser411Leufs*17)	Pathogenic	21	Juvenile; adenomatous polyps	HHT: brain & lung arteriovenous malformations
	Exon 9; MH2 domain	Deletion, frameshift	c.1231_1232del	p.(Ser411Leufs*17)	Pathogenic	46	Juvenile; adenomatous polyps	
	Exon 9; MH2 domain	Deletion, frameshift	c.1245_1248del	p.(Asp415Glufs*20)	Pathogenic	37	Juvenile; hyperplastic polyps	
	Exon 9; MH2 domain	Deletion, frameshift	c.1245_1248del	p.(Asp415Glufs*20)	Pathogenic	29	Juvenile polyps	
	Exon 9; MH2 domain	Deletion, frameshift	c.1245_1248del	p.(Asp415Glufs*20)	Pathogenic	32	Juvenile; adenomatous polyps	Goitre
	Exon 9; MH2 domain	Deletion, frameshift	c.1245_1248del	p.(Asp415Glufs*20)	Pathogenic	24	Juvenile; hamartomatous polyps	
	Exon 9; MH2 domain	Deletion, frameshift	c.1245_1248del	p.(Asp415Glufs*20)	Pathogenic	39	Hyperplastic; adenomatous polyps	
	Exon 9; MH2 domain	Deletion, frameshift	c.1247_1250del	p.(Arg416Lysfs*19)	Unclassified	44	Juvenile polyps	HHT: telangiectasia
	Exon 10; MH2 domain	Deletion, frameshift	c.1343_1367del	p.(Gln448Leufs*20)	Pathogenic	45	Juvenile polyps	
	Exon 10; MH2 domain	Deletion, frameshift	c.1343_1367del	p.(Gln448Leufs*20)	Pathogenic	47	Juvenile; hyperplastic polyps	
	Exon 10; MH2 domain	Deletion, frameshift	c.1343_1367del	p.(Gln448Leufs*20)	Pathogenic	45	Inflammatory, adenomatous, hyperplastic, juvenile polyps	
	Exon 11; MH2 domain	Substitution, missense	c.1573A > G	p.(Ile525Val)	Conflicting interpretations of pathogenicity	57	Adenomatous; hyperplastic polyps	
	Exon 11; MH2 domain	Substitution, missense	c.1573A > G	p.(Ile525Val)	Conflicting interpretations of pathogenicity	50	Adenomtaous; hyperplastic polyps	
**Howe et al. (2004) **[48]	Exon 4; Linker domain	Deletion; premature stop codon	c.608del	p.(Pro203Hisfs*38)	Pathogenic	N/A	Familial JPS	
	Exon 8; MH2 domain	Substitution, missense	c.989A > G	p.(Glu330Gly)	Likely pathogenic	N/A	Familial JPS	
	Exon 8; MH2 domain	Deletion; premature stop codon	c.1037del	p.(Pro346Leufs*38)	Pathogenic	N/A	JPS	
	Exon 8; MH2 domain	Substitution, missense	c.1054G > A	p.(Gly352Arg)	Pathogenic	N/A	Familial JPS	
	Exon 8; MH2 domain	Substitution, missense	c.1081C > G	p.(Arg361Gly)	Likely pathogenic	N/A	Familial JPS	
	Exon 8; MH2 domain	Substitution, missense	c.1081C > G	p.(Arg361Gly)	Likely pathogenic	N/A	Familial JPS	
	Exon 9; MH2 domain	Substitution, nonsense	c.1162C > T	p.(Gln388*)	Pathogenic	N/A	Familial JPS	
	Exon 9; MH2 domain	Deletion; premature stop codon	c.1245_1248del	p.(Asp415Glufs*20)	Pathogenic	N/A	Familial JPS	
	Exon 9; MH2 domain	Deletion; premature stop codon	c.1245_1248del	p.(Asp415Glufs*20)	Pathogenic	N/A	Familial JPS	
	Exon 9; MH2 domain	Deletion; premature stop codon	c.1245_1248del	p.(Asp415Glufs*20)	Pathogenic	N/A	Familial JPS	
	Exon 9; MH2 domain	Deletion; premature stop codon	c.1245_1248del	p.(Asp415Glufs*20)	Pathogenic	N/A	Familial JPS	
	Exon 10; MH2 domain	Deletion; premature stop codon	c.1343_1365del	p.(Gln448Argfs*38)	Pathogenic	N/A	JPS	
	Exon 11; MH2 domain	Substitution, missense	c.1529G > T	p.(Gly510Val)	Pathogenic	N/A	JPS	
	Exon 11; MH2 domain	Deletion; premature stop codon	c.1588del	p.(His530Thrfs*7)	Pathogenic	N/A	Familial JPS	
**Friedl et al. (2002) **[34]	Exon 9; MH2 domain	Deletion, frameshift	c.1245_1248del	p.(Asp415Glufs*20)	Pathogenic	35	JPS with gastric polyposis & gastrectomy at age 35	
	Exon 9; MH2 domain	Deletion, frameshift	c.1245_1248del	p.(Asp415Glufs*20)	Pathogenic	4	JPS with nil extracolonic anomalies and FHx	Lennox disease, macrocephaly, severe psychomotoric retardation
	Exon 9; MH2 domain	Substituiton, missense	c.1157G > A	p.(Gly386Asp)	Pathogenic/Likely pathogenic	10	JPS	HHT features including: pulmonary arteriovenous fistulae, finger clubbing, skeletal thorax abnormalities
	Exon 6	Deletion	c.831_832del	p.(Pro278*)	Pathogenic	12	JPS with massive polyposis at 28y with no gastric polyps	Asymptomatic ventricular septal defect
	Exon 10; MH2 domain	Substitution; missense	c.1342C > T	p.(Gln448*)	Pathogenic	12	JPS	Nil extracolonic features
	Exon 11; MH2 domain	Deletion, frameshift	c.1544del	p.(Arg515Asnfs*22)	Pathogenic	39	JPS with gastric polyposis, giant gastric folds & gastrectomy at 40y	
	Exon 11; MH2 domain	Insertion, frameshift	c.1547_1550dup	p.(Ser517Argfs*11)	Unclassified	21	JPS with gastric polyposis at age 36 & gastrectomy at 40y	
**Handra-Luca et al. (2005) **[41]	Exon 2; MH1 domain	Duplication, frameshift	c.375_381dup	p.(Val128Cysfs*17)	Pathogenic	N/A	JPS	
	Exon 2; MH1 domain	Duplication, frameshift	c.375_381dup	p.(Val128Cysfs*17)	Pathogenic	N/A	JPS	
	Exon 9; MH2 domain	Substitution, nonsense	c.1236C > G	p.(Tyr412*)	Pathogenic	N/A	JPS	
	Exon 9; MH2 domain	Deletion, frameshift	c.1242del	p.(Asp415Thrfs*21)	Pathogenic	N/A	JPS	
	Exon 9; MH2 domain	Deletion, frameshift	c.1245_1248del	p.(Asp415Glufs*20)	Pathogenic	N/A	JPS	
	Exon 9; MH2 domain	Deletion, frameshift	c.1268del	p.(Gly423Glufs*13)	Pathogenic	N/A	JPS	
	Exon 10; MH2 domain	Substitution, nonsense	c.1333C > T	p.(Arg445*)	Pathogenic	N/A	JPS	
	Exon 11; MH2 domain	Substitution, missense	c.1571G > T	p.(Trp524Leu)	Pathogenic	N/A	JPS	
	Exon 11; MH2 domain	Insertion, frameshift	c.1607dup	p.(Asp537Argfs*40)	Pathogenic	N/A	JPS	
**Jelsig et al. (2016)** [55]	Exon 9; MH2 domain	Substitution, missense	c.1156G > A	**p.(Gly386Ser)**	Likely pathogenic	**48**	JPS with FHx, 3 colorectal adenomatous polyps and multiple duodenal polyps	HHT features: epistaxis, telangiectasia, pulmonary and GI AVM, anaemia, stroke
	Exon 9; MH2 domain	Substitution, missense	c.1156G > A	**p.(Gly386Ser)**	Likely pathogenic	**27**	Unconfirmed JPS with FHx and 1 caecal inflammatory polyp	HHT features: epistaxis, telangiectasia, pulmonary AVM
	Exon 9; MH2 domain	Substitution, missense	c.1156G > A	**p.(Gly386Ser)**	Likely pathogenic	**16**	Unconfirmed JPS with FHx and 1 colonic juvenile polyp	HHT features: epistaxis, telangiectasia, pulmonary AVM
	Exon 8; MH2 domain	Substitution. Missense	c.1081C > T	p.(Arg361Cys)	Pathogenic	3	JPS with > 50 juvenile polyps throughout colon, 1 jejunal adenomatous polyp, nil FHx	HHT features: epistaxis, telangiectasia, pulmonary and GI AVM, anaemia, cyanosis, digital clubbing
	Exon 8; SMAD4 activation domain	Deletion, frameshift	c.939del	p.(Ile314Phefs*22)	Pathogenic (unconfirmed)	21	JPS with < 10 colorectal polyps and several ileal polyps; diagnosis of CRC (21) & gastric cancer (37)	HHT features: epistaxis, telangiectasia, anaemia Passed away at 40y
	Exon 10; MH2 domain	Deletion, frameshift	c.1325_1326del	p.(Gln442Leufs*51)	Pathogenic (unconfirmed)	**13**	JPS with FHx and 3 colorectal juvenile polyps	HHT features: epistaxis, telangiectasia, pulmonary AVM, aortopathy (aortic root dilated 5 cm)
	Exon 10; MH2 domain	Deletion, frameshift	c.1325_1326del	p.(Gln442Leufs*51)	Pathogenic (unconfirmed)	**17**	JPS with FHx, > 50 juvenile polyps throughout GIT	HHT features: epistaxis, telangiectasia
	Exon 10; MH2 domain	Deletion, frameshift	c.1325_1326del	p.(Gln442Leufs*51)	Pathogenic (unconfirmed)	**60**	JPS with FHx, > 50 juvenile polyps throughout GIT; diagnosis of CRC (48)	HHT features: epistaxis, telangiectasia
	Exon 10; MH2 domain	Deletion, frameshift	c.1325_1326del	p.(Gln442Leufs*51)	Pathogenic (unconfirmed)	**64**	JPS with FHx, < 10 colorectal juvenile polyps	
	Exon 11; MH2 domain	Duplication, frameshift	c.1587dup	p.(His530Thrfs*47)	Pathogenic	3	JPS with > 50 juvenile polyps throughout GIT	Anaemia
	Exon 1; MH1 domain	Deletion, frameshift	c.1421del	p.(Ser474*)	Pathogenic	**21**	JPS with FHx, > 10–30 colorectal juvenile polyps and several small bowel polyps; diagnosis of CRC (35)	Anaemia
	Exon 1; MH1 domain	Deletion, frameshift	c.1421del	p.(Ser474*)	Pathogenic	**43**	JPS with FHx, > 50 colorectal juvenile polyps and several hyperplastic small bowel hyperplastic polyps	
	Exon 1; MH1 domain	Deletion, frameshift	c.1421del	p.(Ser474*)	Pathogenic	**15**	JPS with FHx, 10–30 colorectal juvenile polyps	
	Exon 5; Linker domain	Insertion, frameshift	c.692dup	p.(Ser232Glnfs*3)	Pathogenic	18	JPS with > 50 colorectal juvenile polyps	Anaemia
**Woodford-Richens et al. (2000) **[108]	Exon 4; Linker domain	Deletion; premature stop codon	c.517_527del	p.(Ser173Thrfs*12)	Pathogenic (unconfirmed)	12	JPS with > 50 sigmoid and recal polyps, FHx of CRC	
	Exon 8; MH2 domain	Substitution, missense	c.1083C > T	p.( =)	Unclassified	16	JPS with 4 colorectal polyps, FHx of CRC & JPS	
	Exon 11; MH2 domain	Deletion; premature stop codon	c.1564_1565del	p.(Pro522Leufs*4)	Pathogenic	N/A	JPS with FHx, > **100 gastric polyps**, 8 colorectal polyps	
	Exon 11; MH2 domain	Deletion; premature stop codon	c.1564_1565del	p.(Pro522Leufs*4)	Pathogenic	N/A	JPS with FHx, gastric JPs	
	Exon 11; MH2 domain	Deletion	c.189_197del	***p.(Asn64_Ala66del)***	Pathogenic (unconfirmed)	N/A	JPS with FHx, extensive polyposis throughout GIT, colectomy at 45y	
	Exon 11; MH2 domain	Deletion	c.189_197del	***p.(Asn64_Ala66del)***	Pathogenic (unconfirmed)	N/A	JPS with FHx, colorectal juvenile polyps, coloectomy at 21y	
	Exon 10; MH2 domain	Substitution; nonsense	c.1333C > T	p.(Arg445*)	Pathogenic	39	JPS with FHx, colonic polyps with tubular adenoma	
	Exon 10; MH2 domain	Substitution; nonsense	c.1333C > T	p.(Arg445*)	Pathogenic	6	JPS with FHx, > 100 juvenile polyps throughout GIT	
**Aretz et al. (2007) **[4]	Exon 9; MH2 domain	Deletion, frameshift	c.1245_1248del	p.(Asp415Glufs*20)	Pathogenic	41	Familial JPS with multiple juvenile colonic and gastric polyps	
	Exon 9; MH2 domain	Deletion, frameshift	c.1245_1248del	p.(Asp415Glufs*20)	Pathogenic	4	Familial JPS with numerous juvenile, hyperplastic and adenomatous colonic polyps	
	Exon 9; MH2 domain	Substitution, missense	c.1157G > A	p.(Gly386Asp)	Pathogenic/Likely pathogenic	10	JPS with > 50 colonic juvenile polyps	
	Exon 6; SMAD4 activation domain	Deletion	c.831_832del	p.(Pro278*)	Pathogenic	12	Familial JPS with > 50 colonic and gastric juvenile, hyperplastic polyps	
	Exon 6–11	Deletion	c.788-?_1659 + ?del	p.?	Unclassified	12	Familial JPS with numerous colonic and gastric juvenile/adenomatous polyps	
	Exon 10; MH2 domain	Substitution, nonsense	c.1342C > T	p.(Gln448*)	Pathogenic	12	JPS with massive juvenile and adenomatous colonic polyps	
	Exon 11; MH2 domain	Deletion	c.(?_-17093)_(*6575_?)del	p.0	Pathogenic (unconfirmed)	18	JPS with > 100 juvenile, adenomatous, hyperplastic colonic polyps	
	Exon 11; MH2 domain	Deletion, frameshift	c.1544del	p.(Arg515Asnfs*22)	Pathogenic	40	JPS with multiple juvenile colonic and gastric polyps	
	Exon 11; MH2 domain	Duplication, frameshift	c.1547_1550dup	p.(Ser517Argfs*11)	Pathogenic (unconfirmed)	24	Familial JPS with numerous juvenile and adenomatous colonic/gastric polyps	
	Exon 8; MH2 domain	Substitution	c.1139G > A	p.(Arg380Lys)	Likely pathogenic	38	JPS with 10 adenomatous colonic polyps with hundreds of gastric juvenile polyps	
	Exon 11; MH2 domain	Deletion	c.(?_-17093)_(*6575_?)del	p.0	Pathogenic (unconfirmed)	33	JPS with massive juvenile and adenomatous colonic polyps	
	Exon 3; Linker domain	Substitution	c.425-6A > G	p.( =)	Uncertain significance	10	Familial JPS with numerous colonic/gastric polyps	
	Exon 11; MH2 domain	Deletion	c.(?_-17093)_(*6575_?)del	p.0	Pathogenic (unconfirmed)	45	Familial JPS with > 30 juvenile, adenomatous and inflammatory colonic polyps and pronounced gastric polyps	
	Exon 9; MH2 domain	Deletion, frameshift	c.1245_1248del	p.(Asp415Glufs*20)	Pathogenic	48	Familial JPS with multiple colonic juvenile polyps and pronounced gastric polyposis	
	Exon 10; MH2 domain	Deletion, frameshift	c.1421del	p.(Ser474*)	Pathogenic	54	Familial JPS with several juvenile colonic and gastric polyps	
	Exon 5–11	Deletion	c.668-?_1659 + ?del	p.?	Unclassified	50	JPS with massive juvenile gastric polyposis with 2 colonic polyps	
	Exon 3; Linker domain	Substitution, nonsense	c.437 T > A	p.(Leu146*)	Pathogenic	48	Familial JPS with multiple juvenile gastric polyps and > 5 colonic polyps	
	Exon 9; MH2 domain	Deletion, frameshift	c.1245_1248del	p.(Asp415Glufs*20)	Pathogenic	23	JPS with > 20 colonic juvenile and inflammatory polyps	
	Exon 8; MH2 domain	Substitution, missense	c.1087 T > C	p.(Cys363Arg)	Pathogenic	5	JPS with multiple colonic polyps	
	Exon 8; MH2 domain	Substitution, missense	c.1082G > A	p.(Arg361His)	Pathogenic	11	JPS with > 50 colonic juvenile polyps	
	Exon 8; MH2 domain	Substitution, missense	c.1081C > T	p.(Arg361Cys)	Pathogenic	3	JPS with > 30 colonic juvenile polyps	
	Exon 11; MH2 domain	Deletion	c.(?_-17093)_(*6575_?)del	p.0	Pathogenic (unconfirmed)	12	JPS with massive juvenile colonic polyposis	
	Exon 2; MH1 domain	Substitution, nonsense	c.403C > T	p.(Arg135*)	Pathogenic	48	JPS with > 20 colonic juvenile polyps and pronounced gastric polyposis	
**Gallione et al. (2004) **[36]	Exon 11; MH2 domain	Deletion	c.1612_1625del	p.(Glu538Hisfs*34)	Pathogenic	**41**	Caecal juvenile polyps, GI cancer	HHT features: telangiectasia, epistaxis, pulmonary and liver AVMs
	Exon 11; MH2 domain	Deletion	c.1612_1625del	p.(Glu538Hisfs*34)	Pathogenic	**8**	Colorectal juvenile polyps	HHT features: telangiectasia, epistaxis, anaemia
	Exon 8; MH2 domain	Substitution, missense	c.1054G > A	***p.(Gly352Arg)***	Pathogenic	**9**	Colorectal juvenile polyps	HHT features: telangiectasia, pulmonary AVM, anaemia, digital clubbing
	Exon 8; MH2 domain	Substitution, missense	c.1054G > A	***p.(Gly352Arg)***	Pathogenic	**5**	Colorectal juvenile polyps, GI cancer	HHT features: pulmonary AVM, anaemia, digital clubbing, stroke with intracranial bleed
	Exon 8; MH2 domain	Substitution, missense	c.1054G > A	***p.(Gly352Arg)***	Pathogenic	**3**	Colorectal juvenile polyps	HHT features: liver AVM, anaemia, digital clubbing
	Exon 11; MH2 domain	Substitution, missense	c.1598 T > G	**p.(Leu533Arg)**	Pathogenic	**15**	Colorectal juvenile polyps	HHT features telangiectases, epistaxis, anaemia
	Exon 11; MH2 domain	Substitution, missense	c.1598 T > G	**p.(Leu533Arg)**	Pathogenic	**14**	Colorectal juvenile polyps	HHT features: telangiectases, epistaxis, anaemia
	Exon 11; MH2 domain	Substitution, missense	c.1598 T > G	**p.(Leu533Arg)**	Pathogenic	**3**	Colorectal juvenile polyps	HHT features: telangiectases, epistaxis, anaemia, digital clubbing, pulmonary & liver AVM
	Exon 11; MH2 domain	Substitution, missense	c.1598 T > G	**p.(Leu533Arg)**	Pathogenic	**N/A**	Not examined	HHT features: pulmonary AVM, anaemia
	Exon 8; MH2 domain	Substitution, missense	c.1081C > G	p.(Arg361Gly)	Likely pathogenic	14	Colorectal and gastric juvenile polyps	HHT features: telangiectases, epistaxis, anaemia
	Exon 9; MH2 domain	Substitution, missense	c.1157G > A	**p.(Gly386Asp)**	Pathogenic/Likely pathogenic	**10**	Colorectal & duodenal juvenile polyps, GI cancer	HHT features: anaemia, pulmonary AVM, telangiectasia, epistaxis, stroke, digital clubbing, stroke, intracranial bleed
	Exon 9; MH2 domain	Substitution, missense	c.1157G > A	**p.(Gly386Asp)**	Pathogenic/Likely pathogenic	**4**	Colorectal juvenile polyps	HHT features: anaemia, pulmonary AVM, digital clubbing, cyanosis
	Exon 11; MH2 domain	Deletion	c.1594del	p.(Ala532Profs*5)	Pathogenic	11	Colorectal juvenile polyps	HHT features: anaemia, telangiectases, epistaxis
	Exon 11; MH2 domain	Substitution, nonsense	c.1600C > T	p.(Gln534*)	Pathogenic	10	Upper GI especially duodenal juvenile polyps, GI cancer	HHT features: liver AVM, epistaxis, digital clubbing, mild seizures
**Gallione et al. (2006) **[37]	Exon 11; MH2 domain	Deletion-insertion Frameshift mutation	c.1596_1597delinsT	p.(Leu533Serfs*4)	Pathogenic	37	Multiple colonic juvenile polyps with GI bleeding	HHT features: telangiectases, epistaxis, AVMs in the lung and GIT, nil FHx
	Exon 8; MH2 domain	Substitution, missense	c.1081C > T	p.(Arg361Cys)	Pathogenic	39	Unclassified regarding JP symptoms	HHT features: telangiectases, epistaxis, pulmonary AVM, liver shunts nil FHx
	Exon 8; MH2 domain	Substitution, missense	c.1081C > T	p.(Arg361Cys)	Pathogenic	13	Mulitple hamartomatous polyps found in the ascending colon + duodenum; Signet ring cell type CRC (caecum)	HHT features: telangiectases, epistaxis, hepatic AVM, anaemia
**Hattem et al. (2008) **[104]	Exon 8; MH2 domain	Substitution, missense	c.970 T > C	p.(Cys324Arg)	Uncertain significance	N/A	JPS	(Juvenile polyposis registry)
	Exon 8; MH2 domain	Substitution, missense	c.989A > G	p.(Glu330Gly)	Likely pathogenic	N/A	JPS	
	Exon 8; MH2 domain	Deletion – > frameshift	c.971del	p.(Cys324Phefs*12)	Pathogenic	N/A	JPS	
	Exon 9; MH2 domain	Substitution, nonsense	c.1193G > A	p.(Trp398*)	Pathogenic	N/A	JPS	
	Exon 10; MH2 domain	Deletion – > frameshift	c.1411_1435del	p.(Gly471Leufs*25)	Pathogenic	N/A	JPS	
	Exon 11; MH2 domain	Duplication – > frameshift	c.1586_1587dup	p.(His530Tyrfs*8)	Pathogenic	N/A	JPS	
	Exon 1–11	Hemizygous deletion	c.(?_-538)_(*6575_?)del	p.0	Pathogenic (unconfirmed)	N/A	JPS	
**Pyatt et al. (2006) **[86]	Exon 2; MH1 domain	Substitution, missense	c.403C > T	p.(Arg135*)	Pathogenic	35	> 20 juvenile polyps	
	Exon 3; Linker domain	Deletion, premature stop codon	c.430_431del	p.(Ser144Argfs*7)	Pathogenic	Infancy	> 5 juvenile polyps in rectum, stomach	
	Exon 5; Linker domain	Insertion, premature stop codon	c.731_732insGCCC	p.(Gln245Profs*20)	Pathogenic (unconfirmed)	9	> 140 juvenile polyps in colon, terminal ileum	
	Exon 7; Linker domain	Duplication, premature stop codon	c.925_928dup	p.(Phe310Cysfs*13)	Pathogenic	17	> 10 juvenile polyps in transverse, descending, sigmoid & rectum	
	Exon 8; MH2 domain	Insertion, premature stop codon	c.982dup	p.(Tyr328Leufs*3)	Pathogenic	9.5	> 9 juvenile polyps—hepatic flexure, rectum	
	Exon 8; MH2 domain	Deletion	c.1088_1090del	p.(Cys363del)	Pathogenic	11	Colonic polyps	HHT symptoms
	Exon 8; MH2 domain	Substitution, missense	c.1091 T > G	p.(Leu364Trp)	Pathogenic	2.5	> 15 juvenile colonic polyps	
	Exon 8; MH2 domain	Deletion, premature stop codon	c.1113del	p.(His371Glnfs*13)	Pathogenic	33	Multiple juvenile polyps in colon and stomach	
	Exon 9; MH2 domain	Deletion, premature stop codon	c.1245_1248del	p.(Asp415Glufs*20)	Pathogenic	10	> 6 juvenile polyps in sigmoid	
	Exon 9; MH2 domain	Deletion, premature stop codon	c.1245_1248del	p.(Asp415Glufs*20)	Pathogenic	21	Innumerable juvenile polyps in colon & stomach	
	Exon 10; MH2 domain	Deletion, premature stop codon	c.1361_1364del	p.(Ala454Glufs*21)	Pathogenic	23	> 20 juvenile polyps in caecum, ascending, descending, sigmoid	
	Intron 10	Intronic	c.1308 + 1G > A	p.( =)	Pathogenic/Likely pathogenic	30	> 15 juvenile polyps with focal adenomatous changes in the colon	
	Exon 11; MH2 domain	Deletion, premature stop codon	c.1596del	p.(Leu533Serfs*4)	Pathogenic (unconfirmed)	7	Colectomy at 7yo	HHT symptoms
**O'Malley et al. (2011)** [80]	Exon 9; MH2 domain	Deletion	c.1245_1248del	p.(Asp415Glufs*20)	Pathogenic	N/A	JPS	HHT features: telangiectasia, pulmonary AVM
	Exon 9; MH2 domain	Deletion	c.1245_1248del	p.(Asp415Glufs*20)	Pathogenic	N/A	JPS	HHT features: FHx, telangiectasia, cranial AVM
	Exon 9; MH2 domain	Deletion	c.1245_1248del	p.(Asp415Glufs*20)	Pathogenic	N/A	JPS	HHT features: FHx. GI AVM
	Exon 9; MH2 domain	Deletion	c.1245_1248del	p.(Asp415Glufs*20)	Pathogenic	N/A	JPS	HHT features: telangiectasia, epistaxis, asthma
	Exon 9; MH2 domain	Deletion	c.1245_1248del	p.(Asp415Glufs*20)	Pathogenic	N/A	JPS	HHT features: telangiectasia, epistaxis, pulmnary AVM, asthma
	Exon 9; MH2 domain	Deletion	c.1245_1248del	p.(Asp415Glufs*20)	Pathogenic	N/A	JPS	HHT features: FHx, telangiectasia, epistaxis, pulmonary AVM, asthma
	Exon 9; MH2 domain	Deletion	c.1245_1248del	p.(Asp415Glufs*20)	Pathogenic	N/A	JPS	HHT features: epistaxis, cranial/pulmonary AVM, asthma
	Exon 9; MH2 domain	Deletion	c.1245_1248del	p.(Asp415Glufs*20)	Pathogenic	N/A	JPS	HHT features: FHx, epistaxis, pulmonary AVM, asthma
	Exon 3/4; Linker domain	Substitution; missense	c.1363C > A	***p.(Gln455Lys)***	Unclassified	N/A	JPS	HHT features: FHx, pulmonary AVM, asthma
	Exon 3/4; Linker domain	Substitution; missense	c.1363C > A	***p.(Gln455Lys)***	Unclassified	N/A	JPS	HHT features: telangiectasia, epistaxis, pulmonary AVM
	Exon 2/3; MH1/Linker	Deletion	c.430_431del	p.(Ser144Argfs*7)	Pathogenic	N/A	JPS	HHT features: N/A
	Exon 8; MH2 domain	Duplication	c.956-?_1139 + ?dup	**p.?**	Unclassified	N/A	JPS	HHT features: telangiectasia, epistaxis, cranial/pulmonary AVM, asthma
	Exon 8; MH2 domain	Duplication	c.956-?_1139 + ?dup	**p.?**	Unclassified	N/A	JPS	HHT features: FHx, telangiectasia, pulmonary AVM
	Exon 9; MH2 domain	Deletion	c.1228_1229del	p.(Gln410Glufs*18)	Pathogenic	N/A	JPS	HHT features: telangiectasia, epistaxis, pulmonary AVM, asthma
	Exon 9; MH2 domain	Deletion	c.1228_1229del	p.(Gln410Glufs*18)	Pathogenic	N/A	JPS	HHT features: FHx, telangiectasia, epistaxis, pulmonary AVM
	Exon 9; MH2 domain	Deletion	c.1228_1229del	p.(Gln410Glufs*18)	Pathogenic	N/A	JPS	HHT features: FHx, telangiectasia, epistaxis, pulmonary AVM, asthma
	Exon 9; MH2 domain	Deletion	c.1228_1229del	p.(Gln410Glufs*18)	Pathogenic	N/A	JPS	HHT features: FHx, epistaxis, cranial/pulmonary AVM
	Exon 9; MH2 domain	Deletion	c.1228_1229del	p.(Gln410Glufs*18)	Pathogenic	N/A	JPS	HHT features: FHx, epistaxis, pulmonary AVM, asthma
	Exon 9; MH2 domain	Deletion	c.1228_1229del	p.(Gln410Glufs*18)	Pathogenic	N/A	JPS	HHT features: FHx, epistaxis
	Exon 9; MH2 domain	Deletion	c.1228_1229del	p.(Gln410Glufs*18)	Pathogenic	N/A	JPS	HHT features: FHx, epistaxis, GI AVM
	Exon 9; MH2 domain	Unclassified	Exon 9	0	Unclassified	N/A	JPS	HHT features: telangiectasia, epistaxis, pulmonary/cranial/GI AVM
**Gallione et al. (2010) **[35]	Exon 2; MH1 domain	Substitution, nonsense	c.302G > A	p.(Trp101*)	Pathogenic	13.5	Colonic and rectal juvenile polyps; JPS	HHT features: pulmonary AVM. Other: Williams syndrome, dental caries, acanthosis nigricans
	Exon 5; Linker domain	Duplication, frameshift	c.692dup	p.(Ser232Glnfs*3)	Pathogenic	22	Colonic, duodenal and gastric juvenile polyps; JPS with FHx	HHT features: pulmonary AVM, FHx of HHT Other: UC, mild HTN
	Exon 8; MH2 domain	Substitution, missense	c.988G > A	p.(Glu330Lys)	Pathogenic	6.5	Colonic and caecal juvenile polyps; JPS with FHx	HHT features: FHx, anaemia, epistaxis
	Exon 8; MH2 domain	Substitution, missense	c.1055G > A	p.(Gly352Glu)	Pathogenic	65	Colonic, duodenal and gastric juvenile polyps; JPS	HHT features: anaemia, pulmonary AVM, telangiectasia, epistaxis
	Exon 8; MH2 domain	Substitution, missense	c.1081C > T	p.(Arg361Cys)	Pathogenic	9	Colonic and gastric juvenile polyps; JPS	HHT features: anaemia, pulmonary AVM, telangiectasia, epistaxis, asthma
	Exon 8; MH2 domain	Substitution, missense	c.1081C > T	p.(Arg361Cys)	Pathogenic	17	GI juvenile polyps; JPS with FHx	HHT features: pulmonary AVM, epistaxis, digital clubbing, FHx of HHT, stroke
	Exon 8; MH2 domain	Substitution, missense	c.1082G > T	p.(Arg361Leu)	Pathogenic	34	Colonic and rectal juvenile polyps; JPS with FHx of CRC (34y)	HHT features: pulmonary AVM, telangiectasia, epistaxis, digital clubbing, FHx of HHT; other: polycythaemia, dental caries, brain abscess (35y)
	Exon 8; MH2 domain	Substitution, missense	c.1082G > A	p.(Arg361His)	Pathogenic	35	Colonic and caecal juvenile polyps; JPS with FHx of CRC (35y)	HHT features: anaemia, pulmonary AVM, telangiectasia, epistaxis; Other: hysterectomy/oophorectomy (36), died of metastatic CRC
	Exon 8; MH2 domain	Substitution, missense	c.1091 T > G	p.(Leu364Trp)	Pathogenic	2.5	Colorectal and caecal juvenile polyps; JPS with FHx	HHT features: pulmonary and hepatic AVM, epistaxis, digital clubbing, FHx of HHT; Other: thrombosis, FTT
	Exon 8; MH2 domain	Deletion, frameshift	c.1102_1103del	p.(Ser368Glnfs*9)	Pathogenic	17	Colonic juvenile polyps; JPS	HHT features: anaemia, telangiectases, epistaxis
	Exon 9; MH2 domain	Substitution, missense	c.1148 T > A	p.(Ile383Lys)	Conflicting interpretations of pathogenicity	21	Colonic, ileal, duodenal juvenile polyps; JPS	HHT features: anaemia, pulmonary AVM, telangiectasia, epistaxis, digital clubbing; other: cyanosis, MV prolapse, asthma
	Exon 9; MH2 domain	Substitution, missense	c.1157G > A	p.(Gly386Asp)	Pathogenic/Likely pathogenic	15	Colonic juvenile polyps; JPS with CRC (20y)	HHT features: anaemia, pulmonary AVM, telangiectasia, epistaxis
	Exon 9; MH2 domain	Deletion, frameshift	c.1245_1248del	p.(Asp415Glufs*20)	Pathogenic	44	Colonic juvenile polyps; JPS with FHx	HHT features: pulmonary AVM, telangiectasia, epistaxis, FHx of HHT
	Exon 11; MH2 domain	Duplication, frameshift	c.1586_1587dup	p.(His530Tyrfs*8)	Pathogenic	3	Colorectal juvenile polyps; JPS	HHT features: anaemia, pulmonary and cerebral AVM, telangiectasia, epistaxis, digital clubbing; other: asthma
	Exon 11; MH2 domain	Substitution, missense	c.1598 T > C	p.(Leu533Pro)	Pathogenic	36	Colonic juvenile polyps; JPS	HHT features: anaemia, pulmonary AVM, telangiectasia, epistaxis, digital clubbing, FHx of HHT
**Duan et al. (2019) **[29]	Exon 2; MH1 domain	Substitution, missense	c.290G > T	**p.(Arg97Leu)**	Conflicting interpretations of pathogenicity	**24**	Nil JPS, HHT, Myhre syndrome	Heritable thoracic aortic disease (HTAD)—ascending aortic dissection at 24y, passed away with ovarian cancer at 44y
	Exon 2; MH1 domain	Substitution, missense	c.290G > T	**p.(Arg97Leu)**	Conflicting interpretations of pathogenicity	**41**	Nil JPS, HHT, Myhre syndrome	HTAD—ascending aortic aneurysm at 41y with bicuspid aortic valve
	Exon 1; MH1 domain	Substitution, missense	c.70A > G	p.(Met24Val)	Uncertain significance	37	Nil JPS, HHT, Myhre syndrome	Type A aortic dissection at 37y; DNA samples
	Exon 5; Linker domain	Substitution, missense	c.736C > A	p.(Pro246Thr)	Uncertain significance	54	Nil JPS, HHT, Myhre syndrome	Type A aortic dissection at 54y
**Rohlin et al. (2017) **[88]	Exon 8; MH2 domain	Deletion, frameshift	c.1110_1114del	p.(His371Aspfs*5)	Pathogenic (unconfirmed)	N/A	Unexplained adenomatous polyposis Pathogenic variant for CRC	
**Calva-Cerqueira et al. (2009) **[20]	Exon 2; MH1 domain	Insertion, frameshift	c.373_374insAT	p.(Ser125Asnfs*5)	Pathogenic	N/A	Sporadic JPS	
	Exon 4; Linker domain	Deletion, frameshift	c.608del	p.(Pro203Hisfs*38)	Pathogenic	N/A	Familial JPS	
	Exon 8; MH2 domain	Substitution, missense	c.989A > G	p.(Glu330Gly)	Likely pathogenic	N/A	Sporadic JPS	
	Exon 8; MH2 domain	Substitution, gene deletion	c.1139 + 1G > A	p.( =)	Pathogenic	N/A	Sporadic JPS	
	Exon 8; MH2 domain	Deletion, frameshift	c.1037del	p.(Pro346Leufs*38)	Pathogenic	N/A	Sporadic JPS	
	Exon 8; MH2 domain	Substitution, missense	c.1054G > A	p.(Gly352Arg)	Pathogenic	N/A	Familial JPS	
	Exon 8; MH2 domain	Substitution, missense	c.1081C > A	p.(Arg361Ser)	Pathogenic	N/A	Sporadic JPS	
	Exon 8; MH2 domain	Substitution, missense	c.1081C > G	p.(Arg361Gly)	Likely pathogenic	N/A	Sporadic JPS	
	Exon 9; MH2 domain	Substitution, nonsense	c.1162C > T	p.(Gln388*)	Pathogenic	N/A	Familial JPS	
	Exon 9; MH2 domain	Deletion, frameshift	c.1245_1248del	p.(Asp415Glufs*20)	Pathogenic	N/A	Sporadic JPS	
	Exon 9; MH2 domain	Deletion, frameshift	c.1245_1248del	p.(Asp415Glufs*20)	Pathogenic	N/A	Familial JPS	
	Exon 9; MH2 domain	Deletion, frameshift	c.1245_1248del	p.(Asp415Glufs*20)	Pathogenic	N/A	Familial JPS	
	Exon 9; MH2 domain	Deletion, frameshift	c.1245_1248del	p.(Asp415Glufs*20)	Pathogenic	N/A	Familial JPS	
	Exon 9; MH2 domain	Deletion, frameshift	c.1245_1248del	p.(Asp415Glufs*20)	Pathogenic	N/A	Sporadic JPS	
	Exon 9; MH2 domain	Deletion, frameshift	c.1245_1248del	p.(Asp415Glufs*20)	Pathogenic	N/A	Sporadic JPS	
	Exon 9; MH2 domain	Substitution, missense	c.1321C > A	p.(Arg441Ser)	Unclassified	N/A	Familial JPS	
	Exon 10; MH2 domain	Deletion, frameshift	c.1343_1365del	p.(Gln448Argfs*38)	Pathogenic	N/A	Sporadic JPS	
	Exon 11; MH2 domain	Substitution, missense	c.1525 T > A	p.(Trp509Arg)	Pathogenic	N/A	Sporadic JPS	
	Exon 11; MH2 domain	Substitution, missense	c.1529G > T	p.(Gly510Val)	Pathogenic	N/A	Sporadic JPS	
	Exon 11; MH2 domain	Deletion, frameshift	c.1588del	p.(His530Thrfs*7)	Pathogenic	N/A	Sporadic JPS	
**Sayed et al. (2002) **[91]	Exon 4; Linker domain	Deletion	c.608del	p.(Pro203Hisfs*38)	Pathogenic	N/A	Familial JPS, > 10 LGI polyps, FHx of GI cancer, nil upper GI involvement	
	Exon 8; MH2 domain	Substitution	c.989A > G	p.(Glu330Gly)	Likely pathogenic	N/A	Familial JPS, FHx of GI cancer	
	Exon 8; MH2 domain	Deletion	c.1037del	p.(Pro346Leufs*38)	Pathogenic	N/A	Unclassified	
	Exon 8; MH2 domain	Substitution	c.1054G > A	p.(Gly352Arg)	Pathogenic	N/A	Familial JPS, > 10 LGI polyps, FHx GI cancer, upper GI involvement	
	Exon 9; MH2 domain	Substitution	c.1162C > T	p.(Gln388*)	Pathogenic	N/A	Familial JPS, > 10 LGI polyps, FHx GI cancer, upper GI involvement	
	Exon 9; MH2 domain	Deletion	c.1245_1248del	p.(Asp415Glufs*20)	Pathogenic	N/A	Familial JPS, > 10 LGI polyps, FHx GI cancer, upper GI involvement	
	Exon 9; MH2 domain	Deletion	c.1245_1248del	p.(Asp415Glufs*20)	Pathogenic	N/A	Familial JPS, > 10 LGI polyps, FHx GI cancer, upper GI involvement	
	Exon 9; MH2 domain	Deletion	c.1245_1248del	p.(Asp415Glufs*20)	Pathogenic	N/A	Familial JPS, FHx GI cancer, nil upper GI involvement	
	Exon 11; MH2 domain	Deletion	c.1588del	p.(His530Thrfs*7)	Pathogenic	N/A	Familial JPS, > 10 LGI polyps, FHx GI cancer, nil upper GI involvement	
**Kim et al. (2000) **[62]	Exon 8; MH2 domain	Substitution; missense	c.1082G > A	p.(Arg361His)	Pathogenic	16	Familial JPS with FHx, > 20 colorectal juvenile polyps, with polypectomy	
	Exon 9; MH2 domain	Substitution; nonsense	c.1162C > T	p.(Gln388*)	Pathogenic	20	JPS with nil FHx, many small bowel, colorectal juvenile polyps, with total colectomy with ileorectal anastomosis	
	Exon 9; MH2 domain	Substitution; missense	c.1168G > A	p.(Glu390Lys)	Pathogenic	16	JPS with nil FHx, 28 colorectal juvenile polyps, with restorative proctocolectomy with ileal pouch-anal anastomosis	
**Roth et al. (1999) **[89]	Exon 4; Linker domain	Substitution; nonsense	c.533C > G	p.(Ser178*)	Pathogenic	38	Sporadic JPS with 10–50 juvenile polyps in stomach, small & large intestines	Congenital panhypopituitarism
	Exon 9; MH2 domain	Substitution; missense	c.1058A > C	**p.(Tyr353Ser)**	Pathogenic	N/A	Familial JPS with FHx, < 10 colonic juvenile polyps; Colorectal (42) & pancreatic carcinoma (50)	
	Exon 9; MH2 domain	Substitution; missense	c.1058A > C	**p.(Tyr353Ser)**	Pathogenic	N/A	Familial JPS with FHx, 10–50 colonic juvenile polyps	
	Exon 10; MH2 domain	Deletion, frameshift	c.1245_1248del	p.(Asp415Glufs*20)	Pathogenic	N/A	Familial JPS with FHx, < 10 colonic juvenile polyps	Adenomatous changes
	Exon 10; MH2 domain	Deletion, frameshift	c.1245_1248del	p.(Asp415Glufs*20)	Pathogenic	N/A	Familial JPS with FHx, > 50 colonic juvenile polyps	Adenomatous changes
	Exon 10; MH2 domain	Deletion, frameshift	c.1245_1248del	p.(Asp415Glufs*20)	Pathogenic	N/A	Familial JPS with FHx, > 50 colonic juvenile polyps; Colorectal carcinoma (30y)	Adenomatous changes
	Exon 10; MH2 domain	Deletion, frameshift	c.1245_1248del	p.(Asp415Glufs*20)	Pathogenic	N/A	Familial JPS with FHx, < 10 colonic juvenile polyps	Adenomatous changes
	Exon 10; MH2 domain	Deletion, frameshift	c.1245_1248del	p.(Asp415Glufs*20)	Pathogenic	N/A	Familial JPS with FHx, 10–50 juvenile polyps in stomach, small and large intestine	Adenomatous changes
**Friedl et al. (1999) **[33]	Exon 8; MH2 domain	Deletion; frameshift	c.831_832del	p.(Pro278*)	Pathogenic	12	Familial JP with > 50 juvenile polyps by age 12. Severe gastric polyposis at 28y. FHx of CRC (father, several paternal relatives)	
	Exon 10; MH2 domain	Deletion; frameshift	c.1245_1248del	p.(Asp415Glufs*20)	Pathogenic	35	Familial JP with multiple juvenile polyps in colon and stomach (35-40y), colonic complications less pronounced. Massive gastric polyposis, requiring partial or total gastrectomy in family members	
	Exon 10; MH2 domain	Deletion; frameshift	c.1245_1248del	p.(Asp415Glufs*20)	Pathogenic	4	Familial JP with multiple juvenile colonic polyps at age 4-5y in 2 brothers	
**Howe et al. (1998) **[47]	Exon 6; SMAD4 activation domain	Deletion; frameshift	c.815_820del	p.(Arg272_Ala274delinsThr)	Pathogenic (unconfirmed)	6	30–40 colonic juvenile polyps (aged 6) with no FHx of JP	
	Exon 8; MH2 domain	Deletion; frameshift	c.1170_1171del	p.(Cys391*)	Pathogenic (unconfirmed)	N/A	Colonic and gastric juvenile polyps with FHx of GI symptoms	
	Exon 9; MH2 domain	Deletion; frameshift	c.1432_1434del	p.(Ile478del)	Pathogenic (unconfirmed)	N/A	JPS	
	Exon 9; MH2 domain	Deletion; frameshift	c.1432_1434del	p.(Ile478del)	Pathogenic (unconfirmed)	N/A	JPS	
	Exon 9; MH2 domain	Deletion; frameshift	c.1432_1434del	p.(Ile478del)	Pathogenic (unconfirmed)	N/A	JPS	
**Wain et al. (2014) **[106]	Exon 2; MH1 domain	Deletion; frameshift	c.263_267del	p.(Lys88Ilefs*14)	Pathogenic (unconfirmed)	N/A	Mutation shared by 3 families including 6 individuals	
	Exon 2; MH1 domain	Deletion; frameshift	c.373_374insAT	p.(Ser125Asnfs*5)	Pathogenic	N/A	1 reported with JP phenotype	
	Exon 5; Linker domain	Deletion; frameshift	c.692dup	p.(Ser232Glnfs*3)	Pathogenic	N/A	3 individuals shared this mutation, reported with JP phenotype & JP-HHT	
	Exon 5; Linker domain	Deletion; frameshift	c.759del	p.(Phe253Leufs*83)	Pathogenic (unconfirmed)	N/A	1	
	N/A	Intronic	c.1308 + 1G > A	p.( =)	Unclassified	N/A	5 unrelated individuals shared this genotype	
	Exon 8; MH2 domain	Substitution; missense	c.1081C > T	p.(Arg361Cys)	Unclassified	N/A	2 individuals with same mutation, where one was reported with JP and the other JP-HHT	
	Exon 8; MH2 domain	Deletion; frameshift	c.1081del	p.(Arg361Alafs*23)	Pathogenic (unconfirmed)	N/A	1	
	Exon 8; MH2 domain	Substitution; missense	c.1082G > A	p.(Arg361His)	Pathogenic	N/A	3 individuals shared this mutation, some reported with JP, and others JP-HHT	
	Exon 8; MH2 domain	Substitution; nonsense	c.1096C > T	p.(Gln366*)	Pathogenic	N/A	1	
	Exon 9; MH2 domain	Substitution; nonsense	c.1193G > A	p.(Trp398*)	Pathogenic	N/A	1 individual reported with JP	
	Exon 9; MH2 domain	Deletion; frameshift	c.1245_1248del	p.(Asp415Glufs*20)	Pathogenic	N/A	Mutation shared by 4 families in 6 individuals	
	Exon 10; MH2 domain	Deletion; frameshift	c.1245_1248del	p.(Asp415Glufs*20)	Pathogenic	N/A	2 unrelated individuals shared this phenotype	
	Exon 11; MH2 domain	Substitution; missense	c.1525 T > A	p.(Trp509Arg)	Pathogenic	N/A	1 individual reported with JP	
	Exon 1–11	Multi-exonic deletion	c.(?_-538)_(1447 + 1_1448-1)del	p.?	Unclassified	N/A	1	
**CASE REPORTS**								
**Sakurai et al. (2021) **[90]	Exon 5; Linker domain	Substitution; nonsense	c.502G > T	p.(Gly168*)	Likely pathogenic	49	Multiple gastric hyperplastic polyps in all parts of the stomach. Gastric juvenile polyposis with adenocarcinoma treated with laparoscopic total gastrectomy	Presented with anaemia, Hx of appendicectomy at 7, FHx of breast cancer, nil FHx of GI cancer or polyposis
**Poaty et al. (2021) **[84]	Exon 9; MH2 domain	Deletion; frameshift	c.1231_1232del	p.(Ser411Leufs*17)	Pathogenic	25	> 100 colonic juvenile polyps, underwent subtotal colectomy, FHx of colon cancer (paternal)	Presented with long medical history of recurrent diarrhoea since 15, associated with loss of weight, mild abdominal pain, rectal bleeding, anaemia, bloating and intestinal occlusion
**Kopiec et al. (2021) **[63]	Exon 8; MH2 domain	Substitution; missense	c.1082G > A	p.(Arg361His)	Pathogenic	11	¬40 juvenile polyps, mostly colorectal, but also gastric and small bowel, with JPS diagnosis. FHx of JPS and HHT (maternal)	Features of HHT: telangiectasias, cyanosis of lips/nails, pulmonary AVM, pulmonary artery dilatation and tricuspid valve regurgitation, epistaxis; Initially presented with rectal bleeding (first time when 4), recurrent nasal bleeding
**Kang et al. (2021) **[59]	Exon 9; MH2 domain	Deletion; frameshift	c.1146_1163del	p.(His382_Val387del)	Pathogenic	7	30–50 colonic juvenile polyps observed with annual polypectomy, ¬10 jejunal polyps and telangiectases	Features of HHT: telangiectasias, pulmonary AVM, epistaxis, digital clubbing
**Hashimoto et al. (2020) **[42]	Exon 8; MH2 domain	Substitution; missense	c.1081C > T	p.(Arg361Cys)	Pathogenic	9	Multiple colonic & numerous gastric juvenile polyps with FHx of maternal colonic & stomach polyps with early gastric cancer + symptoms of HHT	Features of HHT: epistaxis, telangiectasias, pulmonary, liver and mammary gland AVM, anaemia
**Faisal et al. (2020) **[31]	Not mentioned	Not mentioned	Not mentioned	0	#N/A	7	> 20 colonic, gastric and small bowel juvenile polyps. Diagnosed with small bowel carcinoma at 24, passed away from metastatic disease	Hx of IBD on immunosuppressants (increased cancer risk)
**Chang et al. (2020)** [22]	Not mentioned	Not mentioned	Not mentioned	0	#N/A	32	> 100 colonic, small bowel and gastric juvenile polyps, large fungating gastric mass (benign hyperplastic tissue) with no known family history. Underwent total gastrectomy	Presented with severe symptomatic anaemia
**Aagaard et al. (2020) **[1]	Intron b/n exons 5–6 Linker/MH2 domain (breakpoint)	Chromosomal translocation via chromosomal analysis	t(1;18)(p36.1;q21.1)	p.?	Pathogenic (unconfirmed)	N/A	2 juvenile polyps (gastric) with strong FHx of JPS & HHT (brother: multiple JPs, cousin: multiple JPs, CRC at 33y)	HHT features: epistaxis, telangiectasia, FHx of HHT (mother & brother with epistaxis, telangiectasia, PAVM)
**Inoguchi et al. (2019) **[50]	Exon 9; MH2 domain	Deletion; frameshift	c.1245_1248del	p.(Asp415Glufs*20)	Pathogenic	40	Multiple colonic juvenile polyps with substantial gastric polyposis with FHx of gastric cancer	HHT features: epistaxis, telangiectasia, pulmonary AVM, clubbing; other: aortic aneurysm, fatty degeneration of LV, coronary aneurysm
**Leon et al. (2019) **[28]	Exon 9; MH2 domain	Duplication	c.1213dup	p.(His405Profs*24)	Pathogenic (unconfirmed)	**24**	> 10 colonic juvenile hyperplastic polyps with massive juvenile polyposis of the stomach with high grade dysplasia with total gastrectomy (2017), with FHx of JPS	HHT features: epistaxis, pulmonary AVM
		Duplication	c.1213dup	p.(His405Profs*24)	Pathogenic (unconfirmed)	**47**	> 10 colonic juvenile polyps of adenomatous and hyperplastic nature with massive gastric polyposis with subtotal gastrectomy, with FHx of JPS	HHT features: epistaxis in adolescent years
		Duplication	c.1213dup	p.(His405Profs*24)	Pathogenic (unconfirmed)	**23**	> 20 colonic juvenile hamartomatous polyps, as well as massive gastric polyposis	HHT features: epistaxis in younger years, pulmonary AVM
**O'Malley et al. (2019)** [81]	Exon 9; MH2 domain	Deletion	c.1228_1229del	p.(Gln410Glufs*18)	Pathogenic	13	JPS-HHT syndrome, FHx of JPS	Ovarian mass with moderate ascites – > R) ovarian immature teratoma
**Karnsakul et al. (2018) **[61]	Exon 8; MH2 domain	Substitution; missense	c.1052A > T	p.(Asp351Val)	Uncertain significance	6	> 100 colonic juvenile polyps removed via colonoscopy and polypectomy	Juvenile idiopathic arthritis: presented with fever, poor weight gain, upper and lower limb joint swelling & erythema. Features of HHT: digital clubbing, pulmonary and hepatic AVMs
**Bruceta et al. (2018) **[16]	Exon 9; MH2 domain	Deletion; frameshift	c.1245_1248del	p.(Asp415Glufs*20)	Pathogenic	44	> 100 gastric juvenile polyps, few colorectal polyps	Nil features of HHT; other: embolic strokes from atrial septal aneurysm and patent foramen ovale
**Bishop et al. (2018) **[9]	Exon 8; MH2 domain	Substitution; missense	c.1052A > T	p.(Asp351Val)	Uncertain significance	6	> 50 colonic juvenile polyps with diagnosis of JPS-HHT syndrome	Systemic juvenile idiopathic arthritis: knee/ankle pain with swelling; Hx of being premature, Henoch-Schonlein purpura; HHT features: clubbing, pulmonary and hepatic AVM, aortic dilation
**Wiener et al. (2017) **[107]	Exon 10; MH2 domain	Substitution; nonsense	c.1333C > T	p.(Arg445*)	Pathogenic (unconfirmed)	N/A	Nil JPS, FHx of JPS (colonic polyps)	Aortic root dilation with surgical replacement, R) common iliac artery aneurysm; HHT features: epistaxis, telangiectasia
**Ramos et al. (2016) **[87]	Exon 11; MH2 domain	Substitution; missense	c.1561A > C	p.(Thr521Pro)	Uncertain significance	N/A	Duodenal sessile/pendunculated juvenile polyps throughout, tubular adenoma with low grade dysplasia, severe anaemia; FHx of gastric cancer	Hx of ulcerative colitis with total colectomy and ileostony, HHT features: FHx of HHT, epistaxis, telangiectasia, large angioectasia throughout the stomach (angiodysplasia)
**Kadiyska et al. (2016) **[57]	Exon 11; MH2 domain	Deletion; frameshift	c.?	p.(Arg531Glyfs*6)	Pathogenic (unconfirmed)	**11**	One hyperplastic colonic polyp (11y) w/ rectal bleeds, no GI symptoms since	Features of HHT: FHx of HHT, epistaxis, telangiectasia, pulmonary AVM; Other: embolic stroke (30y)
	Exon 11; MH2 domain	Deletion; frameshift	c.?	p.(Arg531Glyfs*6)	Pathogenic (unconfirmed)	**6**	N/A	Features of HHT: clubbing, cyanosis, pulmonary AVM, FHx of HHT (maternal)
**Burmester et al. (2016) **[18]	Exon 9; MH2 domain	Deletion; frameshift	c.1245_1248del	p.(Asp415Glufs*20)	Pathogenic		Family with 3 cases of JPS (colorectal polyps), 1 case of JPS-Menetrier's disease	5 cases of Menetrier's disease, 7 cases of asthma, 8 cases of epistaxis, 5 cases of GORD, 4 cases of gastritis, 14 cases of migraine headaches
**Brosens et al. (2016)** [15]	Not described	Not described	Not described	p.?	#N/A	30	~ 27 colorectal juvenile/inflammatory polyps and multiple gastric hyperplastic polyps	Neurofibromatosis-1 diagnosed at 2y with café-au-lait spots, axillary freckling, bilateral optic nerve gliomas and cutaneous neurofibromas, and FHx of NF-1
**Soer et al. (2015) **[98]	Not described	Deletion of SMAD4 gene	Not described	p.?	#N/A	40	Multiple gastric, small bowel and colonic adenomatous polyps (15) with low grade dysplasia, had gastrectomy, metastatic GI cancer; FHx of son with duodenal & rectal polyps	
		Deletion of exons 1–8	c.(?_-127–1)_(1139 + 1_1140-1)del	p.?	#N/A	48	Multiple gastric polyps, two colonic inflammatory polyps, with iron deficiency anaemia	
**Ngeow et al. (2015) **[78]	SMAD9; MH1 domain	Substitution; missense	#N/A	#N/A	#N/A	38	~ 30 diffuse colonic polyposis (hamartomatous with adipose or ganglioneuroma proliferation) with family history of paternal diffuse polyposis, CRC with deaths in 40s	
**Lin et al. (2015) **[70]	Exon 10; MH2 domain	Substitution	c.1342C > T	p.(Gln448*)	Pathogenic	14	15 pedunculated/sessile colonic juvenile polyps, integrated into bowel wall	HHT features: epistaxis, telangiectasia, FHx of pulmonary AVMs
**Oliveira et al. (2014) **[79]		Balanced translocation	c.(?_-538)_(*6575_?)del	p.0	Pathogenic (unconfirmed)	10	JPS with multiple colonic juvenile polyps with annual colonoscopies, with tubular adenomas by 11y, and multiple adenomatous colonic polyposis by 13y	Other: facial dysmorphism, intellectual disability, developmental delay, ADHD, corpus callosum agenesis HHT features: epistaxis, brain AVM, telangiectasia (JPS-HHT)
**Teekakirikul et al. (2013) **[103]	Exon 10; MH2 domain	Duplication; frameshift	c.1349_1376dup	p.(Ala460Glyfs*43)	Likely pathogenic	7	Multiple juvenile polyps throughout GI tract with low-grade dysplasia with total abdominal colectomy (11y)	Aortopathy: mild dilation of aortic annulus and aortic root; HHT features: pulmonary AVMs, epistaxis
	Exon 9; MH2 domain	Deletion; frameshift	c.1245_1248del	p.(Asp415Glufs*20)	Pathogenic	6	Colonic juvenile polyps, with partial colectomy and multiple polypectomies	Aortopathy: mild aortic root dilation, family history of sudden cardiac death (acute aortic dissection of father); HHT features: pulmonary and hepatic AVM, epistaxis, FHx of HHT; Other: Marfan syndrome, PFO, myclonic epilepsy, TIA, migraine, sleep apnoea, spondylolisthesis
**Honda et al. (2013) **[44]	Exon 11; MH2 domain	Substitution; nonsense	c.1421C > G	p.(Ser474*)	Pathogenic (unconfirmed)	29	Familial JPS with numerous gastric (predominant) + colonic juvenile polyps, necessitating gastrectomy, family history of gastric polyps and CRC	
	Exon 11; MH2 domain	Substitution; nonsense	c.1421C > G	p.(Ser474*)	Pathogenic (unconfirmed)	57	Familial and generalised JPS with multiple gastric (predominant) + small bowel + colonic juvenile polyps, with history of polypectomies	
**Jee et al. (2013)** [52]	Exon 10; MH2 domain	Substitution; nonsense	c.1239C > G	p.(Tyr413*)	Pathogenic	21	Multiple juvenile polyps in stomach, small bowel & colon, with family history of mother with gastric polyposis & CRC (gastrectomy, right hemicolectomy)	
**Stadler et al. (2012) **[99]	Exon 11; MH2 domain	Insertion	c.1507_1508insATCC	p.(Met503Asnfs*25)	Pathogenic (unconfirmed)	38	Diffuse, severe grape-like gastric hyperplastic polyps, and many large colonic polyps, with total gastrectomy + right hemicolectomy with family history of early onset gastric cancer (death of sister/father in 40s)	
**Schwetz et al. (2012) **[95]	Exon 4; Linker domain	Deletion	c.543del	p.(Ile182Serfs*20)	Pathogenic (unconfirmed)	8	Ileocolic intussusception with massive colonic polyposis w/ total colectomy	Family history: grandmother (52y massive polyposis of stomach with total gastrectomy), 7/9 family members with gastric manifestations, 2/9 gallbladder polyps, 3/9 GI AVMs ?JP-HHT
**Piepoli et al. (2012) **[82]	Exon 9; MH2 domain	Deletion; frameshift	c.1245_1248del	p.(Asp415Glufs*20)	Pathogenic	**4**	Familial JPS with several colonic polyps with tubular adenoma & gastric hyperplastic polyps (with H.pylori infection)	
	Exon 9; MH2 domain	Deletion; frameshift	c.1245_1248del	p.(Asp415Glufs*20)	Pathogenic	**13**	Familial JPS with numerous colonic juveile polyps with tubular adenoma & gastric polyps (with H.pylori infection)	
	Exon 9; MH2 domain	Deletion; frameshift	c.1245_1248del	p.(Asp415Glufs*20)	Pathogenic	**17**	Familial JPS with innumerable gastric polyps (with H.pylori infection), few rectal hyperplastic polyps	Menetrier's gastropathy
**Zimmer et al. (2011) **[110]	Exon 9; MH2 domain	Substitution; missense	c.1157G > A	p.(Gly386Asp)	Pathogenic/Likely pathogenic	10	Diffuse colorectal juvenile polyposis, several gastric polyps, large duodenal juvenile polyps	HHT: Ileal AVM, digital clubbing, pulmonary AVM
**Andrabi et al. (2011) **[2]	Exon 10; MH2 domain	Substitution; nonsense	c.1333C > T	p.(Arg445*)	Pathogenic	**7**	Some colonic hamartomatous polyps	Mild mitral regurgitation, family history of sudden cardiac death, Marfan's syndrome; HHT: epistaxis
			Not tested	**Not tested**	Not tested	**21**	5–10 colonic juvenile polyps	Mild aortic dilatation, severe mitral regurgitation, mitral valve prolapse with "Marfan-like CTD" (passed away at 21—arrhythmia precipitated by mitral valve prolapse with underlying marfanoid features)
	Exon 10; MH2 domain	Substitution; nonsense	c.1333C > T	p.(Arg445*)	Pathogenic	**27**	Colonic polyps	Normal echocardiogram in adulthood
	Exon 10; MH2 domain	Substitution; nonsense	c.1333C > T	p.(Arg445*)	Pathogenic	**5**	Mulitple juvenile colonic polyps with total colectomy (19y)	Mild aortic dilatation, mitral valve prolapse
			Not tested	**Not tested**	Not tested	**43**	Multiple colonic juvenile and some adenomatous polyps with metastatic colorectal cancer (pulmonary metastases)	Aortic dilatation, severe mitral regurgitation (passed away at 43)
**Poletto et al. (2010)** [85]			Not tested	Not tested	Not tested	9	Multiple juvenile polyps throughout the colon with family history of JPS (mother)	HHT: epistaxis, digital clubbing, pulmonary AVM, left-to-right shunt, family history of HHT (epistaxis, haemoptysis, telangiectasia, pulmonary AVM)
**Iyer et al. (2010) **[51]	Exon 9; MH2 domain	Deletion	c.1228_1229del	p.(Gln410Glufs*18)	Pathogenic	24	JPS diagnosed given multiple harmatomatous polyps throughout the colon, with total colectomy	HHT: epistaxis, telangiectasia, pulmonary AVM, brain AVM—HHT
**Pitiliciuc et al. (2008) **[83]	Exon 11; MH2 domain	Substitution; nonsense	c.1527G > A	p.(Trp509*)	Pathogenic	31	Generalised juvenile polyposis: Gastric polyps of hypertrophic and polypoid nature with foveolar hyperplasia, nil colorectal/SB polyps, with family history of CRC and gastric cancer	
	Exon 9; MH2 domain	Deletion	c.1245_1248del	p.(Asp415Glufs*20)	Pathogenic	38	Generalised juvenile polyposis: Multiple colonic hyperplastic polyps w/ hypertrophic and polypoid gastropathy, on the background of villous caecal tumour & right hemicolectomy	Menetrier's disease; Hx of R) ovarian teratoma, uterine fibroma, autoimmune hyperthyroidism, lobular breast carcinoma, FHx of brain and breast cancer
**Shikata et al. (2005) **[97]	Exon 5; Linker domain	Insertion; frameshift	c.692dup	p.(Ser232Glnfs*3)	Pathogenic	24	GJP: Profuse gastric polyposis with small areas of adenocarcinoma, nil intestinal polyps, treated with gastrectomy	HHT: pulmonary AVM; other: paroxysmal VT with cardio defibrillator
**Lamireau et al. (2005) **[64]	Exon 11; MH2 domain	Substitution; nonsense	c.1236C > G	p.(Tyr412*)	Pathogenic	**5**	Several colonic juvenile polyps with adenomatous component, large ileal juvenile polyp caussing intussesception	Hypertrophic osteoarthropathy: digital clubbing of finger and toes (acro-osteolysis of distal phalanges of toes with soft-tissue swelling)
	Exon 11; MH2 domain	Substitution; nonsense	c.1236C > G	p.(Tyr412*)	Pathogenic	**3**	Several colonic juvenile polyps	Hypertrophic osteoarthropathy: digital clubbing of finger and toes (acro-osteolysis of distal phalanges of toes with soft-tissue swelling)
**Burger et al. (2002)** [17]	Exon 9; MH2 domain	Substitution; missense	c.1157G > A	p.Gly386Asp	Pathogenic/Likely pathogenic	11	70 colonic juvenile polyps	HHT: pulmonary AVM, digital clubbing
**Baert et al. (1983) **[7]			#N/A	#N/A	#N/A	15	Juvenile intestinal polyposis involving duodenum and colon	Hypertrophic osteoarthropathy: finger clubbing, pretibial swelling – > extensive bilateral laminated new bone formation of ulna and right radius, hands and feet; HHT: pulmonary AVM

## Discussion

### Genotype–phenotype correlations to JPS

#### Variant hotspot

In SMAD4 + JPS patients, the majority of germline DCVs are in the MH2 domain. Up to 80% of DCVs are located between exon 8 and 11, allowing complex formation and translocation to the nucleus in the TGF-β pathway [74]. Small deletions in this location have caused serious cases of JPS with colonic and gastric juvenile polyposis, particularly c.1245_1248del [34, 48, 86, 91]. In another study, 40% (10/25) harboured a genetic alteration at codon 361 in exon 8 [56].

#### Variant types

Most common variant types among SMAD4 DCVs are missense, deletions and small deletions, resulting in frameshift and premature stop codons [27]. Less common are nonsense, insertions, duplications and intronic mutations [20, 23, 93]. In a study by Jones and colleagues [56], 22/25 patients had missense mutations, 2/25 had frameshift mutations and 1/25 had a nonsense mutation, whereas Aretz and colleagues [4] reported 5/17 nonsense, 6/17 frameshift and 6/17 missense mutations, all found to be pathogenic variants, apart from one VUS (missense mutation c.425_426A > G). Chromosomal translocations are rare causes of JPS, historically requiring chromosomal analysis, as described in a JPS-HHT patient [1] with exons 6–11 deleted. Another case report involved a balanced translocation causing loss of the entire SMAD4 gene in a JPS-HHT patient, with dysmorphic features, intellectual disability, developmental delay, and corpus callosum agenesis [79].

#### Histologic phenotype

JPs with SMAD4 DCVs tend to be more epithelial with high crypt-to-stroma ratio, as compared to BMPR1A, with a tendance to be more stromal with a lower ratio. Despite this, dysplasia was equally common in JPS polyps with either mutation [104]. Polyp phenotype is variable, ranging from sessile to pedunculated, with adenomatous, hyperplastic, and inflammatory polyps described in the literature, especially in BMPR1A + JPS [69, 77].

#### Extracolonic polyposis

Patients with SMAD4 DCVs, especially in the linker and MH2 domains, tend to develop and have a family history of UGI polyps, including the small bowel and stomach [34, 91]. SMAD4 DCVs are associated with higher gastric polyp numbers, massive gastric polyposis, and thus, partial or total gastrectomy and gastric cancer [4, 5, 10, 28, 72]. In most cases, SMAD4 DCVs have been detected in patients with both UGI and LGI polyps, whereas polyps are restricted to LGI and anal canal for BMPR1A [100].

#### Aggressive phenotype and variant correlation

Patients with SMAD4 DCVs can develop a more aggressive GI phenotype, with polyps associated with low-grade adenoma, high grade adenocarcinoma, upper GI location, and presence of malformed vessels within the stroma [41]. This is especially the case in patients with DCVs in exons 8–11, especially c.1245_1248del and c.1421delC, involving massive gastric polyposis and GI cancer [33, 34, 55, 80, 89].

### Genotype–phenotype correlations to cancer

Lifetime risk for development of GI cancers in JPS families in different studies range from 9 to 50%, attenuated by improved surveillance and polypectomies over time. Overall, most SMAD4 + JPS patients with GI cancer had DCVs in the MH2 region [36, 77]. They have a higher incidence of GI cancer than those with BMPR1A. In a study by Aytac and colleagues [6], following regular surveillance and appropriate polypectomies, 4/27 individuals with SMAD4 DCVs developed cancer, in comparison to 0/8 of BMPR1A + JPS patients. In another study by Blatter and colleagues [10], incidence of cancer was also higher in SMAD4 carriers, with 20.5% of patients with GI cancer (26/127), compared to 8.4% (8/94) in BMPR1A carriers (*p* = 0.015).

#### Gastric cancer

As aforementioned, gastric polyposis is more common in SMAD4 carriers with JPS, with gastric cancer risk occurring up to 30% in those with SMAD4 DCVs. 7/17 JPS patients with SMAD4 variants had gastric cancer in a study by Aretz and colleagues [4], compared to 0/13 for BMPR1A carriers. In Blatter and colleagues’ study [10], 7/127 SMAD4 carriers had gastric cancer, and 0/94 in BMPR1A.

#### Colorectal cancer

Colorectal cancer occurs at a similar incidence in both causative genes of JPS, where 15/127 (11.8%) had CRC in SMAD4 carriers, compared to 7/94 (7.4%) in BMPR1A carriers [10]. In another study by Schwenter and colleagues [93], 3/14 (21.4%) SMAD4 + JP-HHT patients developed early onset CRC.

#### Somatic studies

From somatic studies, SMAD4 is not seen to be a driver gene for GI cancer, though 16% of primary colorectal tumours have alterations in SMAD4, and 6% in SMAD2. SMAD4 follows APC mutation and precedes TP53 in CRC development [25]. Loss of SMAD4 expression is associated with worse overall survival in patients with CRC, given associations with metastasis and advanced disease [73].

In 30% of pancreatic cancers, SMAD4 is deleted following inactivation of K-ras, increasing TGF-β expression and creating an environment for tumour progression [74]. It is postulated that SMAD4 mutations do not initiate tumour formation, as germline mutations are not associated with pancreatic tumours, but instead promote metastases via LOH and intragenic mutations [73].

### Genotype–phenotype correlations to genetically related allelic disorders of SMAD4

#### HHT & JPS-HHT syndrome

HHT is an autosomal dominant disorder affecting 1 in 5000 to 10 000 individuals, leading to vascular dysplasia with facial and peripheral telangiectasias, together with arteriovenous malformations (AVM) of lung, central nervous system, and GIT. In JPS-HHT, patients share symptoms of JPS and the full range of HHT features [53].

80–85% HHT patients have DCVs in ENG, ACVRL1 or SMAD4, where the former two encode for endothelial receptors of the TGF-β family, necessary to maintain vascular integrity and angiogenesis [42, 93]. SMAD4 DCVs account for < 2% of HHT patients [50]. It is hypothesised that its genetic loss disrupts the balance regulating vascular remodelling and angiogenesis, as well as communication between TGF-β and BMP signalling pathways, as SMAD4 is common to both [35]. Frequency of pulmonary AVMs and gastric involvement were higher amongst SMAD4 + JPS-HHT patients, than those not due to SMAD4 +  [55, 101]. Such DCVs are mostly found in SMAD4’s MH2 region [38], where up to 80% of SMAD4 + JPS are accompanied by HHT. Particularly prevalent DCVs include c.1228_1229delCA, c.1245_1248del, and missense variants in exon 8 [74, 75, 80].

### Myhre syndrome (MS)

**Fig. 6:**
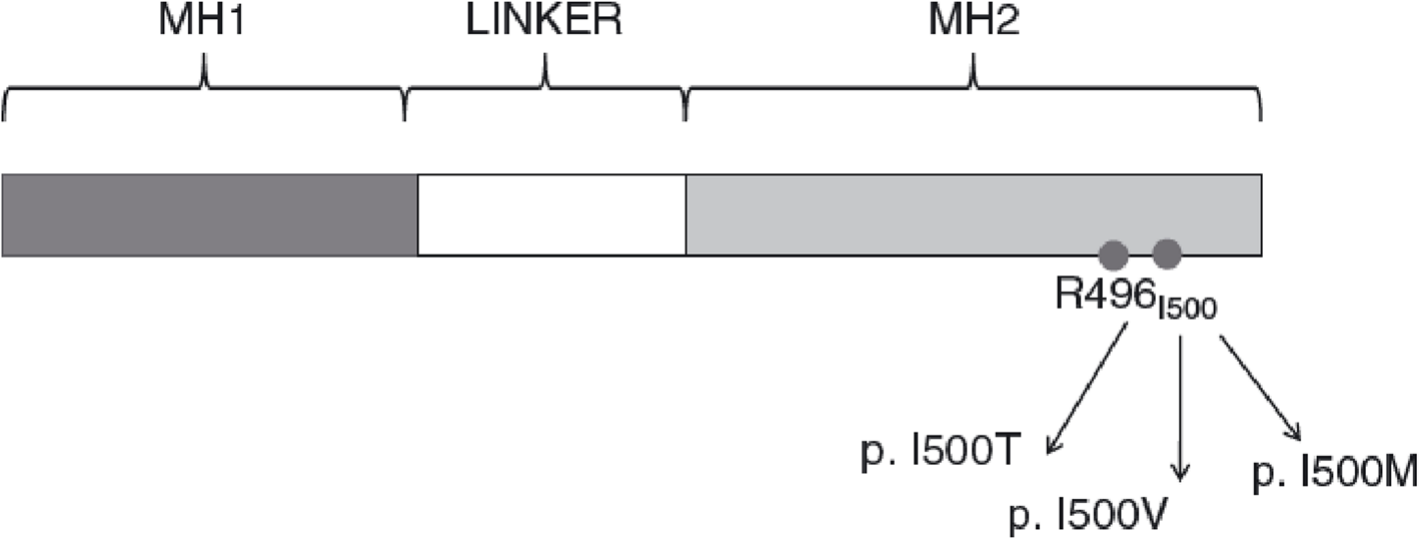
Diagram of variant location in the SMAD4 gene causing MS, mostly around the Ile500 residue in the MH2 domain [67]

**Fig. 7:**
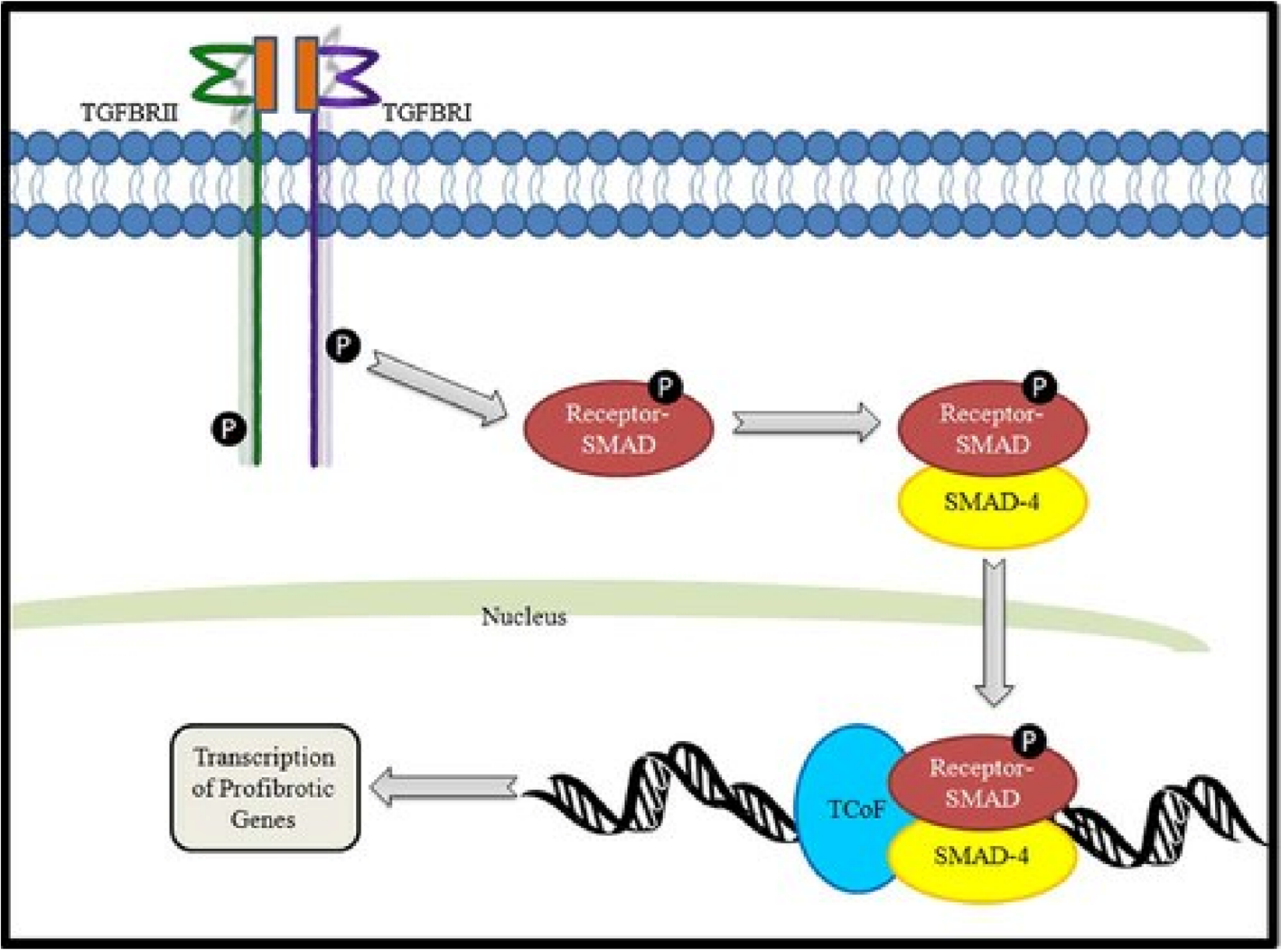
Diagram depicting SMAD4’s involvement in the TGF-β pathway, as shared with TGFB11/2 and FBN1, the signalling pathway to transcribe profibrotic genes [2]

SMAD4 DCVs are solely responsible for MS, a rare developmental disorder with < 100 cases reported. It is characterised by dysmorphic features, joint limitation, muscular pseudohypertrophy, intellectual disability and deafness. DCVs include de novo missense mutations around codon 496–500 in exon 11 (Figs. 6 and 7).

All reported MS cases have occurred independently of JPS [67], with associations to neoplasia. In a recent study, 6/61 MS patients exhibited neoplasia, including endometrial (3/6) and brain tumours (3/6). Given LOF mutations in SMAD4 cause JPS, it is hypothesised that gain-of-function mutations observed in MS may contribute to neoplasia [70].

### Other conditions observed in carriers of SMAD4 variants

#### Cardiac pathologies

Cardiac pathologies have been reported in SMAD4 DCV carriers both independently and in conjunction with JPS-HHT. Manifestations include aortic root dilatation, aneurysm, aortic dissection, and mitral valve dysfunction, including regurgitation and prolapse.

This has been reported in SMAD4 + JPS-HHT patients with variants in the MH2 region, particularly c.1245_1248del and c.1333C > T [2, 16, 103, 106, 107]. Heald and colleagues [43] observed cardiac pathologies in 6/16 SMAD4 + JPS-HHT patients, while Wain and colleagues [106] reported 7/34 JPS patients had connective tissue defects including enlarged aortic root, aortic and mitral valve insufficiency and aortic dissection. Thus, these cardiac pathologies have been postulated to be part of SMAD4-induced HHT manifestations.

Notably, without JPS-HHT, hereditary thoracic aortic disease was described in patients with rare MH1 domain missense mutations. In Duan and colleagues’ case report [29], two family members exhibited different phenotypes, one with ascending aortic dissection, and the other with aortic aneurysm and bicuspid aortic valve. Two unrelated patients both had early onset type A aortic dissection. The proposed pathway is that SMAD4’s involvement in TGF-β signalling is shared with TGFBR1/2 and FBN1, genes involved in connective tissue disorders, where SMAD4 is a transcriptional regulator and tumour suppressor [2].

#### Juvenile idiopathic arthritis (JIA) & Hypertrophic osteoarthropathy (HOA)

In SMAD4 + JPS-HHT patients, JIA has been described in carriers of MH2 domain missense mutations, particularly c.1052A > T in exon 8. Along with colonic JPs with HHT features, patients had upper and lower limb joint swelling, erythema, and digital clubbing [9, 61].

HOA has also been observed in SMAD4 + JPS families, marked by digital clubbing and extensive new bone formation, in MH2 domain DCVs, particularly c.1236C > G [7, 64]. In addition, digital clubbing has been examined in many SMAD4 + JPS-HHT patients, potentially as a manifestation of pulmonary AVMs and right-to-left cardiac shunts [9, 35, 36, 50, 59, 61]. It is postulated that SMAD4 mediates intracellular signals of TGF-β and BMP, found at high levels in bone and cartilage, potentially having a role in bone formation, thus explaining HOA and JIA.

#### Ménétrier’s disease (MD)

MD has been diagnosed concurrently in SMAD4 + JPS patients with gastric polyposis, marked by giant mucosal folds in gastric fundus and body, with diminished acid secretory capacity and protein losing state causing hypoalbuminemia. All MD cases were caused by the SMAD4 variant, c.1245_1248del. In one family, there were 5 MD cases, 3 JPS cases and 1 case of JPS-MD, and two other studies reported familial JPS-MD cases [82, 83]. Mechanistically it is proposed that TGF-alpha overexpression leads to TGF-β pathway inactivation, promoting cell proliferation, where MD could be a manifestation of gastric polyposis in JPS, or be confounding given their similar pathology.

### Diagnosis and genetic testing

SMAD4 + JPS patients mostly have generalised and colonic juvenile polyposis, together with JPS-HHT syndrome in some patients. JPS subtypes include:

- 1. Infantile JP (< 2 years old): rare in SMAD4 + JPS patients, but more common in large deletions involving BMPR1A and PTEN, this is the most severe form of JPS with poor prognosis given aggressive polyp formation [49]
- 2. Generalised JP: JPs throughout the GIT
- 3. Colonic JP or JP coli: JPs exclusively in the colon, common to BMPR1A carriers.
- The latter two phenotypes, generalised and colonic JP, are common to SMAD4, and are caused by DCVs throughout the gene. Malignancy mainly occurs in those with DCVs in the MH2 region, whereas non-malignant polyposis can occur anywhere.

- 4. JPS-HHT syndrome: Exclusive to SMAD4 + patients, JPS-HHT patients have features of both JPS and HHT, caused by DCVs in MH2 region with few exceptions [35, 36].

Once diagnosed with JPS, genetic testing of SMAD4 and BMPR1A germline mutations for probands should occur, in combination with familial genetic counselling [106]. Molecular genetic testing approaches can include BMPR1A and SMAD4 concurrent testing, including multiplex-ligation dependent probe amplification for single or partial gene deletions. Also, serial-gene testing in patients with suspected JPS-HHT can occur, via sequence analysis and gene-targeted duplication or deletion analysis for SMAD4. Contemporarily, multigene panels with BMPR1A, SMAD4, PTEN and other genes, exome and genome sequencing, and chromosomal analysis for translocations are utilised [68, 88].

In addition, all SMAD4 DCV carriers should be screened following JP and HHT protocols, further elucidated in management.

### Implications on management

Surveillance for asymptomatic SMAD4 or BMPR1A DCV carriers, or at-risk family members with no variant detected, are distinct between LGI and UGI tracts. In general, careful surveillance should occur for SMAD4 DCVs in the MH2 domain.

#### LGI management

Asymptomatic LGI surveillance follows conventional endoscopic monitoring, involving 3-yearly full blood examination and colonoscopy from 12–15 years old if no abnormalities are found, or commence screening earlier if symptomatic [24, 109]. Otherwise, if polyps are found, annual screening and endoscopic polyp resection would occur until polyp free [48]. Others suggest patients should be screened annually or biennially regardless, until 70 years old [30]. If colonic polyps are unable to be monitored, controlled or demonstrate malignant potential, this warrants consideration of total abdominal colectomy with ileo-rectal anastomosis or proctocolectomy with or without pouch reconstruction [32, 96]. There are no randomised controlled trials of surveillance to provide a strong evidence base for surveillance and its frequency.

#### UGI management

In terms of UGI surveillance, there are competing thoughts, but are tailored towards known genotype–phenotype correlations. Howe and colleagues [48] suggest UGI endoscopy should take place concomitantly with colonoscopy, in conjunction with biliary and/or pancreatic duct brushings in the context of abnormal liver function tests or elevated amylase. Dunlop [30] advises one or two-yearly UGI endoscopies with colonoscopy from 25 years old. Sayed and colleagues [91] differentiate screening between SMAD4 + JPS patients, who should receive it one to three-yearly, while BMPR1A + or DCV negative patients should be screened five-yearly. Similarly differentiating management based on causative genes, Monahan and colleagues [76] have suggested UGI endoscopic surveillance from 18 years old in SMAD4 + JPS patients, and from 25 years old for BMPR1A + patients, at a frequency of one to three-yearly. Nonetheless, given current data, no UGI surveillance for BMPR1A carriers could be justified due to lack of UGI pathology reported, especially cancer. Additionally, Ménétrier’s disease could be considered during UGI endoscopy. If polyps are detected, UGI endoscopy would be repeated annually with appropriate resection, though complete or partial gastrectomy may be warranted in cases of massive gastric polyposis, dysplasia and gastric cancer, as seen in SMAD4 + JPS patients [40, 66, 97, 98].

#### Extra-intestinal manifestations

SMAD4 + JPS patients should be evaluated for HHT within 6 months of diagnosis, examining for manifestations such as telangiectasia, AVM and digital clubbing. Together with complete blood count, annual targeted clinical examination should occur to monitor for HHT and cardiac pathologies, including full facial observation, peripheral examination to assess for clubbing and joint swelling as per JIA, and cardiorespiratory examination. If HHT is confirmed, screening would thus include 2 yearly bubble contrast echocardiography and pulse oximetry for pulmonary AVMs, followed by CT pulmonary angiogram if abnormal, and a single MRI brain to exclude brain AVMs [32, 35–37, 51].

### Limitations

Overall, limitations of most studies were that they were retrospective, with limited patient numbers given JPS’ rarity, and had incomplete screening of all findings of interest. As a result, low numbers often precluded statistically significant observations. In some cases, there was overrepresentation of SMAD4 + patients given recruitment methods and did not compare phenotypes with other causative genes. As such, larger scale follow-up studies of JPS patients should occur both retrospectively and prospectively to assess genotype–phenotype correlations, with complete screening of all potentially associated syndromes and conditions.

## Conclusion

In conclusion, truncating, missense and nonsense mutations around the MH2 region of SMAD4 are most prevalent and hence more likely to be pathogenic. In SMAD4 + JPS patients, given association with extracolonic polyposis and higher risks of GI cancers, endoscopic screening should occur from 12–15 years at a 3-yearly frequency, especially for patients with DCVs in the MH2 region. With associated genetically related allelic disorders like HHT, cardiac pathologies, HOA and potentially JIA, symptoms should be monitored for these via regular targeted clinical examination. Where HHT is suspected, further investigations should include 2-yearly bubble echocardiogram and a single brain MRI.

This review may help modify clinical diagnosis, screening, surveillance, and management of SMAD4 + JPS patients, as well as aid development of gene specific modifications to the ACMG/AMG criteria for pathogenicity assessment of SMAD4, thus supporting the work of the planned SMAD4 InSiGHT ClinGen Variant Curation Expert Panel.

## Data Availability

The datasets used and analysed during the current study are available from the corresponding author on reasonable request.

## Appendix A

### Search strategy

1. SMAD4-related juvenile polyposis or juvenile polyposis* or infantile juvenile polyposis or juvenile intestinal polyposis or intestinal polyposis or JPS or hamartomatous polyposis syndrome* or retention polyp*
2. SMAD4* or SMAD family member 4 or MADH4 or DPC4 or human SMAD4 protein or JIP or MYHRS or mothers against decapentaplegic homolog 4 or deletion target in pancreatic carcinoma 4 or MAD homolog 4 or deleted in pancreatic carcinoma locus 4 or HSMAD4
3. gene analysis or gene association stud* or genetic association stud* or candidate gene identification or genotype–phenotype correlation* or genotype–phenotype association*
4. genotype* or gene* or genetic* or genom* or phenotype* or DNA* or mutat* or chromosom* or variant
5. 1 AND 2
6. ((SMAD4-related juvenile polyposis or juvenile polyposis* or infantile juvenile polyposis or juvenile intestinal polyposis or intestinal polyposis or JPS or hamartomatous polyposis syndrome* or retention polyp*) adj5 (gene analysis or gene association stud* or genetic association stud* or candidate gene identification or genotype–phenotype correlation* or genotype–phenotype association*))
7. ((SMAD4-related juvenile polyposis or juvenile polyposis* or infantile juvenile polyposis or juvenile intestinal polyposis or intestinal polyposis or JPS or hamartomatous polyposis syndrome* or retention polyp*) adj5 (genotype* or gene* or genetic* or genom* or phenotype* or DNA* or mutat* or chromosom* or variant))
8. 5 OR 6 OR 7

## Abbreviations

JPS: Juvenile polyposis syndrome
DCV: Disease-causing variant(s)
JP: Juvenile polyp
HHT: Hereditary haemorrhagic telangiectasia
AGMG: American College of Medical Genetics and Genomics
GI: Gastrointestinal
LGI: Lower gastrointestinal
UGI: Upper gastrointestinal
VUS: Variants of uncertain significance
TGF-β: Transforming growth factor β
BMP: Bone morphogenetic protein
CRC: Colorectal carcinoma
LOF: Loss-of-function
LOH: Loss-of-heterozygosity
AVM: Arteriovenous malformation
MS: Myhre syndrome
JIA: Juvenile idiopathic arthritis
HOA: Hypertrophic osteoarthropathy
MD: Ménétrier’s disease
IBD: Inflammatory bowel disease
